# Pre- and Post-synaptic Mechanisms of Neuronal Inhibition Assessed Through Biochemically Detailed Modeling of GABA_B_ Receptor Signaling

**DOI:** 10.1523/JNEUROSCI.0544-25.2025

**Published:** 2025-08-06

**Authors:** Tuomo Mäki-Marttunen, Jan Fredrik Kismul, Kadri Pajo, Jan Michael Schulz, Tiina Manninen, Gaute T. Einevoll, Marja-Leena Linne, Ole A. Andreassen, Jeanette Hellgren Kotaleski

**Affiliations:** ^1^Faculty of Medicine and Health Technology, Tampere University, Tampere 33014, Finland; ^2^Department of Biosciences, University of Oslo, Oslo 0316, Norway; ^3^Department of Neuroscience, Karolinska Institutet, Solna 171 77, Sweden; ^4^Department of Biomedicine, University of Basel, Basel 4031, Switzerland; ^5^Department of Physics, Norwegian University of Life Sciences, Ås 1432, Norway; ^6^Department of Physics, University of Oslo, Oslo 0316, Norway; ^7^Centre for Precision Psychiatry, Institute of Clinical Medicine, University of Oslo, Oslo 0424, Norway; ^8^Science of Life Laboratory, Computational Science and Technology, KTH Royal Institute of Technology, Stockholm 100 44, Sweden

**Keywords:** CA1 pyramidal cells, GIRK channels, layer V pyramidal cells, mass-action-law-based modeling, multicompartmental modeling, N-type Ca^2+^ channels, RGS proteins, short-term depression, subcellular-level neuron modelling

## Abstract

GABA_B_ receptors (GABA_B_Rs) are an important building block in neural activity. Despite their widely hypothesized role in many basic neuronal functions and mental disorder symptomatology, there is a lack of biophysically and biochemically detailed models of these receptors and the way they mediate neuronal inhibition. Here, we developed a computational model for the activation of GABA_B_Rs and its effects on the activation of G protein-coupled inwardly rectifying potassium (GIRK) channels as well as inhibition of voltage-gated Ca^2+^ channels. To ensure the generality of our modeling framework, we fit our model to electrophysiological data including patch-clamp and intracellular recordings that described both pre- and postsynaptic effects of the receptor activation. We validated our model using data on postsynaptic effects of GABA_B_Rs on layer V pyramidal cell firing activity ex vivo and in vivo and confirmed the strong impact of dendritic GIRK channel activation on the neuron output. Finally, we reproduced and dissected the effects of a knockout of RGS7 (a G protein signaling protein) on CA1 pyramidal cell electrophysiological properties, which shows the potential of our model in generating insights on genetic manipulations of the GABA_B_R system and related genetic variants. Our model thus provides a flexible tool for biochemically and biophysically detailed simulations of different aspects of GABA_B_R activation that can reveal both foundational principles of neuronal dynamics and brain disorder-associated traits and treatment options.

## Significance Statement

GABAB receptors (GABABRs) play a crucial role in brain function, mediating slow inhibition in neurons. However, their complex mechanisms and interactions with other proteins have been poorly understood. Our study presents a detailed computational model that simulates how GABABRs work, particularly their impact on potassium and calcium channels in neurons. By analyzing real data from experiments, we verified our model’s accuracy and demonstrated how it can predict the effects of genetic changes. This model offers valuable insights into the basic processes that govern brain function and has the potential to guide research into treatments for mental health issues, providing a new avenue for understanding and addressing these complex disorders.

## Introduction

Computational models of neurons and neuronal networks have emerged as a useful tool to obtain knowledge about the central nervous system and how its functions arise as a complex interplay of various neurochemical processes. *γ*-Aminobutyric acid (GABA) receptors of type B (GABA_B_Rs) represent one essential component of neural activity in both cortical and subcortical brain regions (Bettler et al., 2004; Groen, 2011; Tureček et al., 2023). In contrast to the better understood and more extensively targeted ionotropic GABA receptors of type A (GABA_A_Rs; Ghit et al., 2021), GABA_B_Rs are G protein-coupled receptors and thus the inhibition they exert is slower and dependent on a larger intracellular cascade (Bettler et al., 2004). Activation of the GABA_B_R releases the beta–gamma subunit of the G_i_ protein (G_i*βγ*_) that, upon binding to ion channels, inhibits N-type voltage-gated Ca^2+^ channels (VGCCs; typically presynaptic) and activates G protein-coupled inwardly rectifying K^+^ (GIRK) channels (typically postsynaptic; Kohl and Paulsen, 2010). In addition, GABA_B_Rs have been shown to directly inhibit postsynaptic L-type Ca^2+^ channels in some neuron types (Pérez-Garci et al., 2006, 2013; Schulz et al., 2021). The impact of GABA_B_R activation is complex as it also mediates short-term synaptic depression in both excitatory and inhibitory presynaptic terminals (Isaacson et al., 1993; Olpe et al., 1994). This presents a challenge for mechanistic analysis of GABA_B_R-mediated effects in single neurons as well as neural circuits: namely, the effects of manipulations of the GABA_B_R system are difficult to predict and the observed phenomena are difficult to generalize between neuron types (Craig and McBain, 2014). Overcoming this challenge is important due to the association of the GABA_B_R pathway with heritable mental disorders such as schizophrenia (Trubetskoy et al., 2022) and its promise as a drug target (Evenseth et al., 2020). Here, we propose the use of biochemically detailed computational modeling to enhance our understanding of GABA_B_R-mediated effects and to help address this challenge.

Traditionally, computational models of neurons and neuronal networks have heavily relied on GABA_A_R-mediated currents, often excluding GABA_B_R-mediated currents or simplifying them as static, long-tailed inhibitory currents (Traub et al., 1994; Turi et al., 2019). While models of GABA_B_R-mediated inhibition that describe some of the intracellular machinery exist, they only focus on either post (Destexhe, 1998; Park et al., 2014) or presynaptic (Li et al., 2020) effects, and they do not include the contribution of key proteins involved such as regulators of G protein signaling (RGS) proteins. RGS proteins inhibit G-protein activity by hydrolyzing the G_i*α*_-bound guanosine triphosphate (GTP) to guanosine diphosphate, permitting the G_i*α*_ subunit to reassociate with G_i*βγ*_ to inactivate the G_i_ protein (Zhong et al., 2003). Here, we aimed to develop a detailed modeling framework to enable investigations of novel GABA_B_R features not included in current models. We fit a mass-action-law-based model including GABA_B_Rs, G_i_, RGS proteins and GIRK and N-type Ca^2+^ channels to electrophysiological patch-clamp and intracellular recording data (Isaacson et al., 1993; Olpe et al., 1994) and present a biophysically detailed modeling framework in which both pre- and postsynaptic effects of GABA_B_R manipulations can be quantified. We verified the appropriate functioning of the presynaptic depression model using a detailed model of presynaptic terminal integrated into an axon segment and incorporated a neurotransmitter release model. We then validated the postsynaptic model using additional data on effects of GABA_B_R activation on layer 5 pyramidal cell (L5PC) firing under patch-clamp stimulus (Schulz et al., 2021) or electrical paw stimulation (Palmer et al., 2012) and explored the relationship between the effects of pre- and postsynaptic GABA_B_R activation on L5PC firing. We employed our model to dissect the causes of altered excitability and plasticity in CA1 pyramidal cells of RGS7-KO mice (Ostrovskaya et al., 2014). This shows that our model can be used for modeling of genetic manipulations of GABA_B_R pathway and is thus immediately useful for exploring mechanisms and possible treatments of mental disorders.

## Methods

In this work, we utilize two types of computational modeling: (1) biochemically detailed modeling, where the concentration of different molecular species are modeled according to the law of mass action and (2) multicompartmental, biophysically detailed neuron modeling, where neuronal electrophysiology is modeled across the entire neuron. In most of the computational experiments, we combine these two types of modeling using the NEURON simulator.

We first developed a single-compartment model of GABA_B_ receptor activation without incorporating membrane ion-channel mechanisms. To demonstrate its potential in neuroscience research, we integrated it into various multicompartmental Hodgkin–Huxley-type models—Table 1 summarizes the models (both electrophysiological and biochemical) employed in this work. A list of acronyms used in this work is shown in Table 2.

**Table 1.** Summary of models

Model	Number of compartments	References	Where described	Where employed
Single-compartment GABA_B_R activation model	1 or more	(Developed here^a^)	Section “Construction of the GABA_B_R activation mode”	Figures 1–7
Model of an axon and axon terminal (presynaptic)	5	(Developed here^b^)	Section “Presynaptic modeling: composite model of an axon and a GABA_B_R-containing presynaptic terminal”	Figures 2, 4–5
Layer V pyramidal cell model (postsynaptic)	196	Hay et al. (2011)	Section “Postsynaptic modeling: L5PC model and inclusion of GIRK channel electrophysiology”	Figures 3–5
CA1 pyramidal cell model (postsynaptic)	144	Combe et al. (2018)	Section “Postsynaptic modeling: CA1 pyramidal cell model and inclusion of GIRK channel electrophysiology”	Figures 6–7
Neurotransmitter release model (cross-synaptic)	1 or more	(Developed here^c^)	Section “Cross-synaptic modeling: neurotransmitter-gated model of AMPAR- and NMDAR-mediated currents”	Figures 4–5

The models developed in this work (^a^^,^^b^^,^^c^) were based on existing models: a: Zhong et al. (2003), Jedrzejewska-Szmek et al. (2017), Mäki-Marttunen et al. (2020); b: Destexhe et al. (1994), Migliore et al. (1995), Hay et al. (2011); c: (Wang, 1999).

**Table 2.** List of acronyms

AHP	Afterhyperpolarization
AMPA	*α*-Amino-3-hydroxy-5-methyl-4-isoxazolepropionic acid
AMPAR	AMPA receptor
AP	Action potential
DC	Direct current
EE	Excitatory to excitatory
EPSC	Excitatory postsynaptic current
GABA	*γ*-Aminobutyric acid
GABAR	GABA receptor
GABA_A_R	GABAR type A
GABA_B_R	GABAR type B
GDP	Guanosine diphosphate
GIRK	G protein-coupled inwardly rectifying K^+^
GTP	Guanosine triphosphate
HCN	Hyperpolarization-activated cyclic nucleotide-gated
HFS	High-frequency stimulation
IE	Inhibitory to excitatory
IPSC	Inhibitory postsynaptic current
ISI	Inter-stimulus interval
L5PC	Layer 5 pyramidal cell
LFS	Low frequency stimulation
LTD	Long-term depression
LTP	Long-term potentiation
NMDA	*N*-methyl-d-aspartate
NMDAR	NMDA receptor
NSGA	Non-dominated sorting genetic algorithm
NT	Neurotransmitter
PPR	Paired-pulse ratio
RGS	Regulator of G protein signaling
SBML	Systems biology markup language
SD	Standard deviation
SK	Small-conductance calcium-activated K^+^
VGCC	Voltage-gated Ca^2+^ channel
VGSC	Voltage-gated Na^+^ channel

### Construction of the GABA_B_R activation model

#### Modeling intracellular signaling networks underlying G_i*βγ*_ activation and their effects on ion channels

We modeled the intracellular signaling in the GABA_B_R-containing subcellular compartments (presynaptic terminal, postsynaptic spine, or a dendritic compartment) using the law of mass action. We only used deterministic simulations. The reactions as well as the default initial concentrations and reaction rates were adopted from Jedrzejewska-Szmek et al. (2017) and Mäki-Marttunen et al. (2020; apart from the RGS reactions that were taken from Zhong et al., 2003) and are listed in Table 3A.

**Table 3.** The biochemically detailed model of GABA_B_R activation

A		
Reactions	*k*_*f*_	*k*_*b*_
R1: gaba → gabaOut	*k*_1_ × 0.0005	0.0
R2: gaba + GABA_B_R ↔ gabaGABA_B_R	*k*_2_ × 5.555 · 10^−6^	*k*_2*x*_
R3: gaba + GABA_B_RG_i_ ↔ gabaGABA_B_RGi	*k*_2_ × 5.555 · 10^−6^	*k*_2*x*_
R4: GABA_B_R + G_i_ ↔ GABA_B_RGi	*k*_3_ × 7.5 · 10^−5^	*k*_3_ × 0.000125
R5: gabaGABA_B_R + G_i_ ↔ gabaGABA_B_RGi	*k*_3_ × 0.00015	*k*_3_ × 0.00025
R6: gabaGABA_B_RG_i_ → gabaGABA_B_RG_i*βγ*_ + GiaGTP	*k*_3_ × 0.000125	0.0
R7: gabaGABA_B_RG_i*βγ*_ → gabaGABA_B_R + G_i*βγ*_	*k*_3_ × 0.001	0.0
R8: GiaGTP + RGS ↔ GiaGTPRGS	2 · 10^−6^	0.002
R9: GiaGTPRGS → GiaGDP + RGS	0.03	0.0
R10: GiaGDP + G_i*βγ*_ → Gi	0.00125	0.0
R11: GIRK + G_i*βγ*_ ↔ GIRKG_i*βγ*_	*k*_4_ × 1.4 · 10^−5^	*k*_5_ × 0.001
R12: GIRKG_i*βγ*_ + G_i*βγ*_ ↔ GIRKG_i*βγ*_2	*k*_4_ × 1.4 · 10^−5^	*k*_5_ × 0.001
R13: GIRKG_i*βγ*_ 2 + G_i*βγ*_ ↔ GIRKG_i*βγ*_ 3	*k*_4_ × 1.4 · 10^−5^	*k*_5_ × 0.001
R14: GIRKG_i*βγ*_ 3 + G_i*βγ*_ ↔ GIRKG_i*βγ*_ 4	*k*_4_ × 1.4 · 10^−5^	*k*_5_ × 0.001
R15: VGCC + G_i*βγ*_ ↔ VGCCG_i*βγ*_	*k*_4_ × 1.4 · 10^−5^	*k*_5_ × 0.001
B		
Parameter	Lower limit	Upper limit
*k*_1_	0.0	1,000
*k*_2_	0.0001	1,000
*k*_3_	0.0	1,000
*k*_4_	0.0	100
*k*_5_	0.0	100
[RGS_post_]	0.1 μM	10 μM
[RGS_pre,E_]	0.1 μM	10 μM
[RGS_pre,I_]	0.1 μM	10 μM
*f*_GABA_	0	3,000/ms

**A**: Reactions describing the activation of GABA_B_Rs and G_i_ protein and the binding of G_i*βγ*_ to GIRK and N-type VGCCs. The reaction rates were adopted from Jedrzejewska-Szmek et al. (2017) , Mäki-Marttunen et al. (2020), and Zhong et al. (2003): some reaction rates were used as such, while others were multiplied by coefficients *k*_1_, *k*_2_, *k*_3_, *k*_4_, and *k*_5_ that were varied in a multiobjective optimization framework [see (B)]. The unit of *k*_*b*_ is always 1/ms and the unit of *k*_*f*_ is 1/ms or 1/(nM·ms) when the reaction has one or two reactants, respectively. **B**: The ranges for all optimized parameters. The parameters *k*_1_, *k*_2_, *k*_3_, *k*_4_, and *k*_5_ are coefficients for the reaction rates of reactions R1–R7 and R11–R15 [see (A)]. The parameter *k*_2*x*_ was determined so that the affinity was 110 nM as measured experimentally (Lehmann et al., 2009), i.e., *k*_2*x*_ = 5.555·10^−6^/(nM·ms)* × k*_2_  × 110 nM. In addition, the initial concentration of the RGS proteins in all domains and the GABA flux were varied. The concentrations of the rest of the species were [GABA_B_R] = 0.4 μM, [G_i*βγ*_] = 2.6 μM, [GIRK] = 1 μM, and [VGCC] = 0.1 μM, unless otherwise stated. Both the reactions (A) and initial concentrations (B) were the same in pre- and postsynaptic compartments, apart from those of VGCCs (R15), which were absent from postsynaptic compartments, GIRK channels (R11–R14), which were absent from presynaptic compartments, and RGS proteins (R8–R9), whose concentration differed between presynaptic terminals of E→E and I→E synapses and postsynaptic compartments (B). The zero concentration of GIRK channels effectively blocks reactions R11–R14 at the presynaptic terminals while the zero concentration of VGCCs in effect blocks reaction R15 at the postsynaptic compartments.

The binding of G_i*βγ*_ to GIRK (postsynaptic) or N-type Ca^2+^ (presynaptic) channels was considered as the main output of the model. The GIRK channel was activated in a dose-dependent manner by binding of G_i*βγ*_: the conductance was 0 when non-bound and 1%, 6%, 26%, or 100% of the maximal conductance when bound by 1, 2, 3, or 4 G_i*βγ*_ molecules (Dascal and Kahanovitch, 2015). The VGCCs, in turn, were inhibited (only one G_i*βγ*_ molecule per channel was required for this) such that the activation curve was shifted by +10 mV and the activation time constant was made 30% larger in the G_i*βγ*_-bound compared to the non-bound VGCCs (Huynh et al., 2015).

#### Model fitting

To fit the rate coefficients governing the GABA_B_R activation dynamics, we used a multiobjective optimization algorithm, NSGA-II (Deb et al., 2002), as implemented in Bahl et al. (2012) and Mäki-Marttunen et al. (2018). We simultaneously adjusted the model of Table 3 to fit three data sets: (1) GABA_B_R-mediated inhibitory postsynaptic currents (IPSCs; Isaacson et al., 1993), (2) GABA_B_R-mediated short-term synaptic depression of excitatory-to-excitatory (E→E) synapses (Isaacson et al., 1993), and (3) GABA_B_R-mediated short-term synaptic depression of inhibitory-to-excitatory (I→E) synapses (Olpe et al., 1994). All three data sets were obtained by Schaffer collateral stimulation and intracellular recordings from CA1 pyramidal cells (Isaacson et al., 1993; Olpe et al., 1994). We varied the reaction rates for GABA uptake from the extracellular medium (*k*_1_), the forward rate of GABA binding to the GABA_B_R (*k*_2_), a multi-reaction rate coefficient for G_i_ protein activation (*k*_3_), and the forward (*k*_4_) and backward (*k*_5_) reaction rates for G_i*βγ*_ binding to the voltage-gated ion channels, namely, GIRK and N-type Ca^2+^ channel (Table 3B). We also varied the magnitude of the GABA flux into the vicinity of the synapse and the RGS concentrations. All varied parameters applied to all three types of simulations, except for the RGS concentration, which was allowed to vary between the synapse types (postsynaptic E→E, presynaptic E→E, and presynaptic I→E) to capture the differences in the dynamics of GABA_B_R effects (Isaacson et al., 1993; Olpe et al., 1994).

We introduced two objective functions to constrain the model responses according to the electrophysiological data. The first objective function quantified the difference between predicted postsynaptic GIRK conductance and the GABA_B_R-mediated IPSCs measured in Isaacson et al. (1993), both normalized by their maximal values:

$$f_{1}(p)=\sum_{i=1}^{16}\left|\frac{{\mathrm{IPSC}}_{i}}{{\mathrm{IPSC}}_{\max}}-\frac{g_{\mathrm{GIRK}(t_{i};p)}}{\max_{t}(g_{\mathrm{GIRK}}(t;p))}\right|,$$

where *t*_i_, *i* = 1, …, 16 are the time instants of the IPSC measurements and IPSC_*i*_ are the corresponding IPSC values. Here, *g*_GIRK_(*t*; *p*) is the predicted GIRK conductance at time *t* after stimulus onset given model parameters *p*. It was determined from the simulation of postsynaptic sections (no VGCCs, 1 μM GIRK channels, which corresponds to 120 molecules/μm^2^ in a compartment with a diameter of 0.8 μm, and RGS concentration set according to the parameter given for postsynaptic sections) using the predicted concentrations of GIRK channels in different states as

$$g_{\mathrm{GIRK}}(t;p)={\bar{g}}_{\mathrm{GIRK}}\frac{0.01\times [\mathrm{GIRK}G_{i\beta \gamma }](t;p)+0.06\times [\mathrm{GIRK}G_{i\beta \gamma }2](t;p)+0.26\times [\mathrm{GIRK}G_{i\beta \gamma }3](t;p)+[\mathrm{GIRK}G_{i\beta \gamma }4](t;p)}{\left[\mathrm{GIRK}](t;p)+[\mathrm{GIRK}G_{i\beta \gamma }](t;p)+[\mathrm{GIRK}G_{i\beta \gamma }2](t;p)+[\mathrm{GIRK}G_{i\beta \gamma }3](t;p)+[\mathrm{GIRK}G_{i\beta \gamma }4](t;p\right)},\;(1)$$

where ${\bar{g}}_{\mathrm{GIRK}}$ is the theoretical maximum (here, we used the value 33 pS; Chen and Johnston, 2005) for GIRK channel conductance and [GIRKG_i*βγ*_**X**](t;p), **X**=1..4 is the predicted concentration of GIRK channels bound with **X** G_i*βγ*_ molecules at time *t*, given parameters *p*. The second objective function quantified the difference between predicted VGCC binding and the experimentally observed paired-pulse ratios (PPR), both normalized, across the two presynaptic types:

$$f_{2}(p)=\sum_{i=1}^{6}\left|\frac{1-{\mathrm{PPR}}_{i,E}}{\max(1-{\mathrm{PPR}}_{i,E})}-\frac{\left[{\mathrm{VGCC}G_{i\beta \gamma }}_{E}](t_{i};p\right)}{\max_{t}([\mathrm{VGCC}{G_{i\beta \gamma }}_{E}](t;p))}\right|+\sum_{i=1}^{7}\left|\frac{1-{\mathrm{PPR}}_{i,I}}{\max(1-{\mathrm{PPR}}_{i,I})}-\frac{\left[\mathrm{VGCC}{G_{i\beta \gamma }}_{I}](t_{i};p\right)}{\max_{t}([\mathrm{VGCC}{G_{i\beta \gamma }}_{I}](t;p))}\right|,$$

where PPR_*i*,E_ and PPR_*i*,I_ are the PPRs in E→E or I→E synapses as measured in Isaacson et al. (1993) or Olpe et al. (1994), respectively, for the *i*th time point *t*_i_. Here, [VGCCG_i*βγ*E_](*t*; *p*) and [VGCCG_i*βγ*I_](*t*; *p*) are the predicted concentration of G_i*βγ*_-bound VGCC in E→E or I→E synapses, respectively, at time *t*, given parameters *p*. We also introduced a third objective function that maximizes the absolute signal amplitudes in the three types of simulations:

$$\begin{array}{rl} \;f_{3}(p) & =-\max_{t}(0.01\times [\mathrm{GIRK}G_{i\beta \gamma }](t;p)+0.06\times [\mathrm{GIRK}G_{i\beta \gamma }2](t;p)\\ & \;+0.26\times [\mathrm{GIRK}G_{i\beta \gamma }3](t;p)+[\mathrm{GIRK}G_{i\beta \gamma }4](t;p))\\ & \;\times \max_{t}\left([\mathrm{VGCC}{G_{i\beta \gamma }}_{E}](t;p)\right)\times \max_{t}\left([\mathrm{VGCC}{G_{i\beta \gamma }}_{I}](t;p)\right). \end{array}$$

We ran the NSGA-II optimization algorithm with 2,000 sample parameter sets for 25 generations. The third objective function was not used for selection of the final parameter set but as a signal to avoid obtaining models that produce correct G_i*βγ*_ binding dynamics when normalized but little total G_i*βγ*_ binding. We picked the parameter set that performed best in the two first objectives compared to median error values, i.e., *p*_final_ = argmin_*p*_(*f*_1_(*p*)/median_*p*′_(*f*_1_(*p*′)) + *f*_2_(*p*)/median_*p*′_(*f*_2_(*p*′))).

### Presynaptic modeling: composite model of an axon and a GABA_B_R-containing presynaptic terminal

In the simulations of GABA_B_R-mediated short-term synaptic depression, we modeled the presynaptic terminal (axonal bouton) surrounded by axonal compartments. The axonal bouton was modeled as a single-compartment cylinder with 0.8 μm length and 0.4 μm diameter located between two active axon segments with 20 μm length and 1 μm diameter. On both ends of the active axon there were 100 μm long, 1 μm thick passive axon segments. For the active segments, we implemented the active membrane mechanisms and the Ca^2+^ decay mechanism from the active axon model of Hay et al. (2011). For the axonal bouton, we adapted the N-type Ca^2+^ channel model from Migliore et al. (1995) by adding the description of the “reluctant” mode of activity, which simulates the channel behavior under G_i*βγ*_-binding. This was implemented such that a fraction (determined by the intracellular signaling model as $\frac{\left[\mathrm{VGCC}G_{i\beta \gamma }\right]}{\left[\mathrm{VGCC}]+[\mathrm{VGCC}G_{i\beta \gamma }\right]}$) of the channels were in the reluctant mode while the rest ( $\frac{\left[\mathrm{VGCC}\right]}{\left[\mathrm{VGCC}]+[\mathrm{VGCC}G_{i\beta \gamma }\right]}$) remained in the “willing” mode. The activation midpoint was 10 mV higher and the time constant was 31% larger in the reluctant than in the willing mode (Colecraft et al., 2000; Huynh et al., 2015). We assumed the passive axon segments to be completely myelinated and thus void of any transmembrane currents.

We stimulated the end of the (passive) axon by a square-pulse current with amplitude 0.3 nA and 0.5 ms duration to induce an action potential (AP) in the active axon compartments and the axonal bouton. The AP caused a Ca^2+^ influx through the N-type Ca^2+^ channels. We introduced the model of Ca^2+^-induced neurotransmitter release from Destexhe et al. (1994). Namely, we introduced the reactions involving Ca^2+^ binding to a fusion factor molecule and further to a neurotransmitter-containing vesicle to induce the release in the axonal bouton (in addition to the GABA_B_R model)—see Table 4.

**Table 4.** Table of reactions and initial concentrations between molecular species involved in Ca^2+^-induced release of neurotransmitter

Reactions	*k*_*f*_	*k*_*b*_	Initial concentrations
R16: 4 × Ca + Ffactor ↔ FfactorCa4	$50\times 10^{9}\;\frac{1}{\mathrm{ms}\cdot {\mathrm{mM}}^{4}}$	0.1 $\frac{1}{\mathrm{ms}}$	[Ffactor] = 1 μM
R17: FfactorCa4 + Ves ↔ FfactorCa4Ves	100,000 $\frac{1}{\mathrm{ms}\cdot \mathrm{mM}}$	0.1 $\frac{1}{\mathrm{ms}}$	[Ves] = 1 μM
R18: FfactorCa4Ves → 10,000*NT + Ffactor + Ves + 4*Ca	4 $\frac{1}{\mathrm{ms}}$	0	Other species: 0 μM
R19: NT → { }	10 $\frac{1}{\mathrm{ms}}$	0	

### Postsynaptic modeling: L5PC model and inclusion of GIRK channel electrophysiology

We used the Hay model of thick-tufted L5PCs (Hay et al., 2011) to analyze the postsynaptic effects of GABA_B_R activation. The model includes Hodgkin–Huxley-type description of 11 ion-channel mechanisms (namely, the passive leak, transient and persistent Na^+^, transient and persistent K^+^, Kv3.1- and M-type K^+^, SK, HCN, and high- and low-voltage-activated Ca^2+^ currents) and a description of intracellular Ca^2+^ dynamics (Hay et al., 2011). We introduced the GABA_B_R activation model (Table 3) in all of the dendritic sections using the NEURON RxD formalism (McDougal et al., 2013). We then introduced a GIRK current model based on an existing Kir channel model (Stegen et al., 2012; Yim et al., 2015) in these sections and coupled the conductance of this channel to the intracellular signaling model. In the experiments of Figure 3, all dendritic sections contained the GABA_B_R activation model and the interacting GIRK channels, whereas in the experiments of Figures 4 and 5 only those dendritic sections that received synaptic inputs included the GABA_B_R and GIRK models. To model the bath application of the agonist, we introduced an additional backward reaction GABA ← GABA_out_ with the same rate as the forward reaction R1 (Table 3A). In this model, the GIRK current was described as

$$i_{\mathrm{GIRK}}(t)=g_{\mathrm{GIRK}}(t)l(t)(E_{K}-V_{m}(t)),\;(2)$$

where *g*_GIRK_(*t*) is as in Equation 1, *l*(*t*) is a gating variable, *E*_K_ is the reversal potential of K^+^, and *V*_m_ is the membrane potential. The gating variable obeys the differential equation

$$\frac{dl}{dt}=\frac{l_{\infty }(V)-l}{\tau_{l}(V)},\;(3)$$

where $l_{\infty }(V)=\frac{1}{1+\exp(-(-98.92\;\mathrm{mV}-V)/10.89\;\mathrm{mV})}$ and $\tau_{l}=\frac{1\;\;\mathrm{ms}}{0.0061\cdot \exp(-V/67\;\mathrm{mV})+0.082\cdot \exp(V/67\;\mathrm{mV})}$.

### Postsynaptic modeling: CA1 pyramidal cell model and inclusion of GIRK channel electrophysiology

We used the Combe model of CA1 pyramidal cells (Combe et al., 2018) to analyze the postsynaptic GABA_B_R activation in CA1 pyramidal cells and its effects on the cell’s excitability and response to plasticity-inducing stimulation. The model includes Hodgkin–Huxley-type description of 12 ion-channel mechanisms (the passive leak, transient and persistent Na^+^, delayed rectifier K^+^, A- and M-type K^+^, slow- and medium AHP (Ca^2+^-activated) K^+^, L-, R- and T-type Ca^2+^, and HCN currents) and a description of intracellular Ca^2+^ dynamics (Combe et al., 2018; Bianchi et al., 2012). Similar to the L5PCs, we introduced the GABA_B_R activation model (Table 3) in all apical and basal dendritic sections together with the GIRK current model (Eq. 2). When determining the Ca^2+^ currents through the *N*-methyl-d-aspartate (NMDA) receptors, we multiplied the total NMDAR-conducted currents by 0.1 as approximately 10% of NMDAR-conducted were estimated to be Ca^2+^ currents (Burnashev et al., 1995).

### Cross-synaptic modeling: neurotransmitter-gated model of AMPAR- and NMDAR-mediated currents

For the simulations assessing the effects of presynaptic GABA_B_Rs on postsynaptic neuron activity, we integrated the neurotransmitter release model (Table 4), regulated by the GABA_B_R activation model (Table 3), into an existing synaptic model. Namely, we adapted the *α*-amino-3-hydroxy-5-methyl-4-isoxazolepropionic acid (AMPA)/NMDA synapses of Wang (1999) and Mäki-Marttunen and Mäki-Marttunen (2022) to make them activated by the release of neurotransmitter in the axonal bouton in a dose-dependent manner. The AMPAR conductance was described by *g*_AMPA_ = *g*_AMPA,max_*s*_AMPA_ where *s*_AMPA_ was determined by

$$\frac{ds_{\mathrm{AMPA}}}{dt}=-s_{\mathrm{AMPA}}/\tau_{s,\mathrm{AMPA}}+c_{\mathrm{NT}}\cdot [\mathrm{NT}].$$

Here, *τ*_*s*,AMPA_ = 2 ms is the time constant of the decay of the AMPAR-mediated current, [NT] is the concentration of the neurotransmitter (glutamate) in the synaptic cleft and *c*_NT_ [unit 1/(ms mM)] is the constant relating the neurotransmitter concentration to the steepness of the rise of the AMPAR conductance. Similarly, the NMDAR conductance was described by *g*_NMDA_ = *g*_NMDA,max_*s*_NMDA_ where *s*_NMDA_ was determined by

$$\begin{array}{rl} \frac{ds_{\mathrm{NMDA}}}{dt} & =-s_{\mathrm{NMDA}}/\tau_{s,\mathrm{NMDA}}+\alpha_{s,\mathrm{NMDA}}\cdot x_{\mathrm{NMDA}}\cdot (1-s_{\mathrm{NMDA}})\;\mathrm{and}\\ \frac{dx_{\mathrm{NMDA}}}{dt} & =-x_{\mathrm{NMDA}}/\tau_{x,\mathrm{NMDA}}+c_{\mathrm{NT}}\cdot [\mathrm{NT}]. \end{array}$$

Here, *τ*_*x*,NMDA_= 2 ms and *τ*_*s*,NMDA_ = 100 ms are the time constants of the rise and decay, respectively, of the NMDAR-mediated current, and the neurotransmitter gating is mediated by the coupling of [NT] and *x*_NMDA_ in a similar fashion as the coupling of [NT] and *s*_AMPA_.

### Code availability

All simulations were run using NEURON v. 8.2.6, using Python (3.9.20) interface. Our simulation scripts are available at ModelDB (accession number 2018268). To ensure easier model re-use and accordance with the FAIR data principles (Wilkinson et al., 2016), we also provide the single-compartment GABA_B_R biochemical signaling model in SBtab and SBML formats. The model with three experimental settings was manually transferred into human-readable spreadsheet-based SBtab format (Lubitz et al., 2016) from which it was converted into an SBML file via the SBtabVFGEN tool as part of the modeling workflow by Santos et al. (2022).

## Results

### The model fits experimental data on presynaptic short-term depression and postsynaptic GIRK currents

To obtain a realistic and generalizable model of GABA_B_R activation leading to GIRK channel activation and N-type Ca^2+^ channel inactivation, we fit the reaction rates to electrophysiological data relating to both pre- and postsynaptic phenomena. The time course of postsynaptic GIRK channel activity has been measured in CA1 pyramidal cells (Isaacson et al., 1993), where the GABA_B_R-mediated GIRK currents peaked at 200 ms and returned to zero in approximately 800 ms. As for presynaptic effects of GABA_B_R activation in E→E synapses, the same study used a conditioning burst of 5 pulses at 50 Hz followed by a single test pulse to determine the time course of heterosynaptic short-term depression of excitatory postsynaptic currents (Isaacson et al., 1993). This heterosynaptic short-term depression was shown to be GABA_B_R-dependent, and it had a maximal amplitude (minimal ratio between the two stimuli) at approximately 300 ms and returned to baseline (ratio between the two stimuli close to one) at approximately 1,600 ms (Isaacson et al., 1993). Comparable PPR dynamics were measured for the GABA_B_R-dependent short-term depression of inhibitory postsynaptic currents in CA1 pyramidal cells (Olpe et al., 1994). In this work, we assigned the differences between the decay times in GABA_B_R-dependent activity of GIRK or VGCC channels to the prevalence of RGS proteins by allowing the RGS protein concentration to vary between different domains (postsynaptic spine, excitatory presynaptic terminal and inhibitory presynaptic terminal), while we kept the reaction rates equal in all domains.

We applied a multiobjective optimization strategy to constrain our model. We varied nine different model parameters according to the boundary conditions shown in Table 3B. We introduced objective functions to (1) minimize the difference between the experimentally measured GIRK channel current and the GIRK conductance predicted at 16 time points (80–700 ms after stimulus onset), and (2) minimize the summed difference between GABA_B_-dependent PPRs measured for excitatory and inhibitory presynaptic terminals and the PPRs predicted based on beta/gamma subunit of G_i_ binding to VGCCs—see Methods for details. We employed NSGA-II (Deb et al., 2002; Bahl et al., 2012) for 25 generations (population size *N* = 2,000) in 15 independent trials and for each trial picked the parameter sets that resulted in smallest normalized sum of pre- and postsynaptic objective functions.

**Figure 1.**
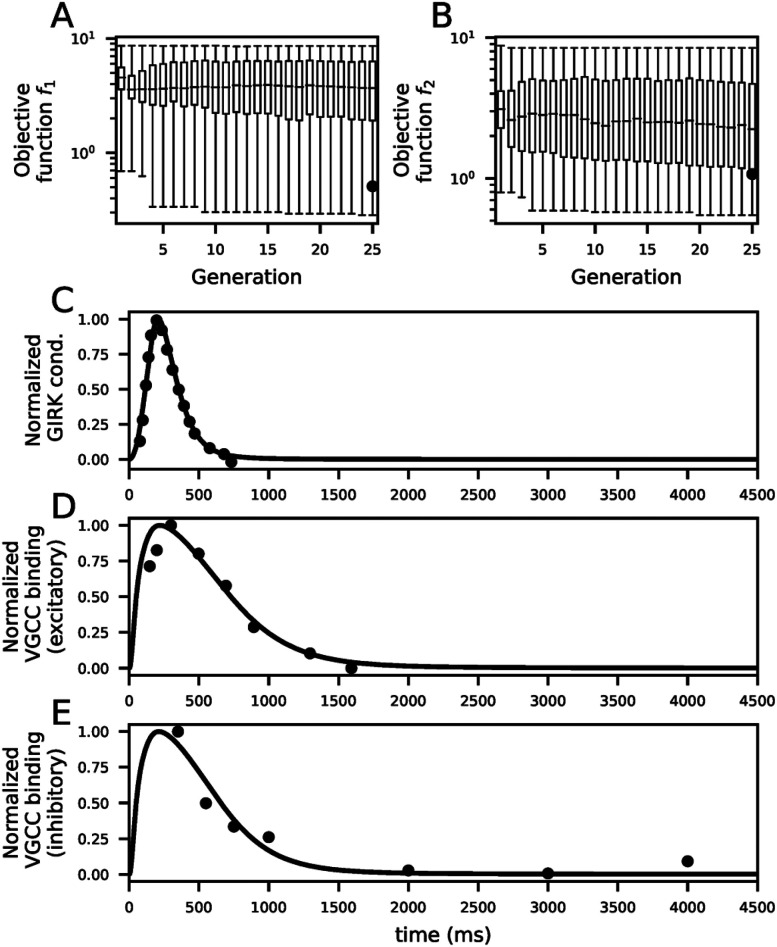
Fitting of the GABA_B_R signaling pathway model. ***A–B***, Evolution of the objective functions *f*_1_ (***A***) and *f*_2_ (***B***) across 25 generations of multi-objective optimization. The parameter set chosen for simulations shown in panels (***C***)–(***E***) first appeared at the final (25th) generation (objective function values denoted by black dots) of the multi-objective optimization—this parameter set had the smallest sum of objective functions normalized by their medians $\frac{\;f_{1}}{{\mathrm{median}}_{p}(f_{1}(p))}+\frac{\;f_{2}}{{\mathrm{median}}_{p}(f_{2}(p))}$. ***C–E***, Time course of the experimental data-derived (dots) and model predicted (solid lines) GIRK conductances at the postsynaptic neuron (***C***), VGCC binding at the presynaptic side of the E→E synapses (***D***), and VGCC binding at the presynaptic side of the I→E synapses (***E***).

The evolution of the objective functions showed little improvement after 10 generations (Fig. 1*A–B*). The best parameter set fit well into both post- (Fig. 1*C*) and presynaptic (Fig. 1*D–E*) data—see Table S1 for the chosen parameter set as well as the best parameter sets obtained from all 15 trials.

Taken together, our model fits electrophysiological data on the GABA_B_R-mediated short-term depression and postsynaptic GIRK conductance dynamics.

### Verification of the presynaptic GABA_B_R model by inclusion of additional components

While the postsynaptic GIRK conductance, as predicted by the model, can be directly related to measured electrophysiological data, the relationship between the predicted binding of the G_i*βγ*_ to the VGCCs and the PPR involves mechanisms not described in the model fitted in Figure 1. Here, we aimed to make sure that the GABA_B_R activation-mediated binding of the G_i*βγ*_ to the VGCCs produced an effect that was rapid and strong enough to dampen the amplitude of the N-type Ca^2+^ currents in a realistic setting that involved the interplay of ionic currents in the presynaptic terminal.

**Figure 2.**
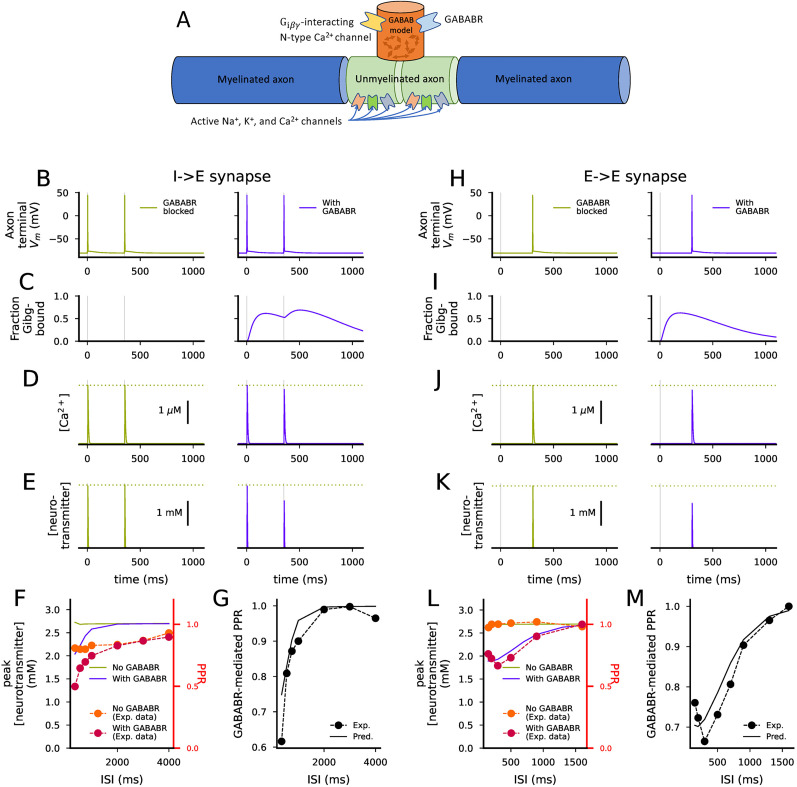
A model of a piece of axon with active ion channels for AP generation and the presynaptic terminal with the intracellular GABA_B_R activation model coupled to N-type VGCCs reproduces the experimental PPR data. ***A***, An illustration of the model of the presynaptic terminal. The model consists of two passive axonal compartments surrounding two active axonal compartments with Na^+^, and K^+^, and Ca^2+^ ion channel models from Hay et al. (2011). A presynaptic terminal with N-type VGCCs (Migliore et al., 1995) coupled to the intracellular GABA_B_R activation model branched from between the active compartments. ***B–D***, The membrane potential (***B***), the fraction of N-type VGCCs in the G_i*βγ*_-bound state (***C***), and the intracellular Ca^2+^ concentration (***D***) at the inhibitory presynaptic terminals with (blue) and without (light green) GABA_B_R activation in response to two stimuli (at 0 and 350 ms) injected at the further end of the passive axon compartment. In the presence of GABA_B_R activation, the first stimulus activates the GABA_B_Rs, leading to inactivation of some of the N-type Ca^2+^ channels in the terminal (***C***, blue), which causes a depression of the Ca^2+^ transient induced by the second stimulus (***D***, blue). ***E***, The neurotransmitter concentration at the synaptic cleft in response to the stimuli in the presence (blue) and absence (light green) of GABA_B_R activation. The depression of the second Ca^2+^ transient induced by the first stimulus leads to less neurotransmitter being released when GABA_B_Rs are present (***E***, blue). ***F***, The peak of the neurotransmitter concentration (mM; right axis) corresponding to the second stimulus with respect to the ISI between the two stimuli. The curves show the predicted peak amplitudes in the presence (blue) and absences (light green) of GABA_B_R activation, and the data points indicate the PPRs (right axis) measured experimentally in the presence (orange) and absence (red) of GABA_B_R antagonist. ***G***, The peak of the neurotransmitter concentration of the GABA_B_R-active case normalized by the corresponding peak in the GABA_B_R-blocked case with respect to the ISI. The data points show the experimentally measured PPR in the GABA_B_R-active case normalized by the corresponding PPR in the GABA_B_R-blocked case. ***H–K***, The experiment of (***B–E***) repeated for excitatory synapses with ISI 300 ms, but the first stimulus (“S1” at 0 s) only activates the GABA_B_Rs in the presynaptic terminal, not the AP generation in the axon. ***L–M***, The experiment of (***F–G***) repeated for excitatory synapses.

We verified the appropriate functioning of the presynaptic model by inserting the biochemical model (Table 3) into a composite model of a piece of axon and a presynaptic terminal containing Hodgkin–Huxley-type description of Na^+^ and K^+^ currents (from Hay et al., 2011) as well as N-type Ca^2+^ currents (from Migliore et al., 1995), where the Ca^2+^ current kinetics were dependent on the binding of the G_i*βγ*_ to the N-type Ca^2+^ channel as reported in Colecraft et al. (2000) and Huynh et al. (2015; Fig. 2*A*). We also incorporated a Ca^2+^-influx-mediated neurotransmitter release (Destexhe et al., 1994). We assumed that the activation of the axon terminal either directly (in the I→E synapse) or indirectly (in the E→E synapse) caused GABA to be released and detected by the GABA_B_Rs in the axon terminal, as suggested by experimental data (Isaacson et al., 1993; Olpe et al., 1994; Scanziani, 2000). To model the stimulation protocol of the heterosynaptic short-term depression of Isaacson et al. (1993), the axon was stimulated only for the second incoming stimulus in the E→E synapse while the first stimulus (“S1” in Isaacson et al., 1993) was assumed to affect other nearby axon terminals that caused the GABA to be released and to activate the GABA_B_Rs in the modeled axon terminal. By contrast, to model the stimulation protocol of Olpe et al. (1994) in the I→E synapse, the modeled axon terminal was activated for both stimuli, and since the GABA_B_Rs were assumed to function as autoreceptors, both stimuli triggered GABA_B_R activation.

In simulations of both I→E and E→E synapses, stimulation of the axon led to an AP in the axon terminal both in presence and absence of GABA_B_R activation (Fig. 2*B,H*). The GABA inputs (indicated by gray vertical lines) caused activation of the presynaptic GABA_B_Rs, leading to G_i*βγ*_ binding to the presynaptic Ca^2+^ channels (Fig. 2*C,I*) and inactivating them. The inclusion of GABA inputs decreased the amplitudes of the presynaptic Ca^2+^ transients (Fig. 2*D,J*) and the amplitude of the neurotransmitter release (Fig. 2*E,K*) in response to the second input (blue), while the neurotransmitter release was as strong for the second input as for the first input in the absence of GABA_B_R activation (green). We repeated these simulations for all inter-stimulus intervals (ISIs) used in experimental setups (Isaacson et al., 1993; Olpe et al., 1994; Scanziani, 2000), and estimated the relative difference in the amplitude of neurotransmitter release between the first and the second electrical stimulation in each case. Although the absolute PPRs estimated as the relation of the neurotransmitter release between the second and first input was mildly overestimated both in presence and absence of GABA_B_R activation in the I→E synapse (Fig. 2*F*), normalization of the GABA_B_R-involving PPR curve by the GABA_B_R-excluding one gave a good fit to the corresponding experimental data (Olpe et al., 1994; Fig. 2*G*). The model of E→E synapse, by contrast, fit both absolute (Fig. 2*L*) and GABA_B_R-normalized (Fig. 2*M*) heterosynaptic short-term depression curves measured in Isaacson et al. (1993).

We ran a series of additional simulations of the composite model to show the validity of our model in describing the GABA_B_R-mediated form of short-term synaptic depression. The results shown in Figure 2 were produced with a representative parameter set out of the 15 tested ones, but the good fit was retained by most of the fitted parameter sets—in only 5/15 models the average error between predicted and measured normalized PPR curve was larger than 0.05 (Fig. S1*A–B*). We also analyzed the behavior of our model under a stimulation protocol (Isaacson et al., 1993) that exhibited short-term facilitation of excitatory postsynaptic currents. Short-term facilitation index (i.e., the amplitude of the second EPSC response normalized that of the first one given two consecutive stimuli) is expected to be increased by processes or manipulations that decrease neurotransmitter release, such as GABA_B_R-mediated inhibition of presynaptic VGCCs (Isaacson et al., 1993). The validity of our biochemically detailed model in describing short-term plasticity was supported by these simulations: our model predicted that the facilitation index (here quantified as the ratio of neurotransmitter peak values) was 13.7% smaller in the absence compared to the presence of GABA_B_R activation while experimental data suggests a decrease of 10.5±2.8% (Isaacson et al., 1993; Fig. S1*C–E*).

Taken together, our model of GABA_B_R-mediated presynaptic short-term depression fits the electrophysiological data also when included in a composite model including a biophysically detailed description of neurotransmitter release.

### The model fitted to AP firing activity profiles from L5PCs

**Figure 3.**
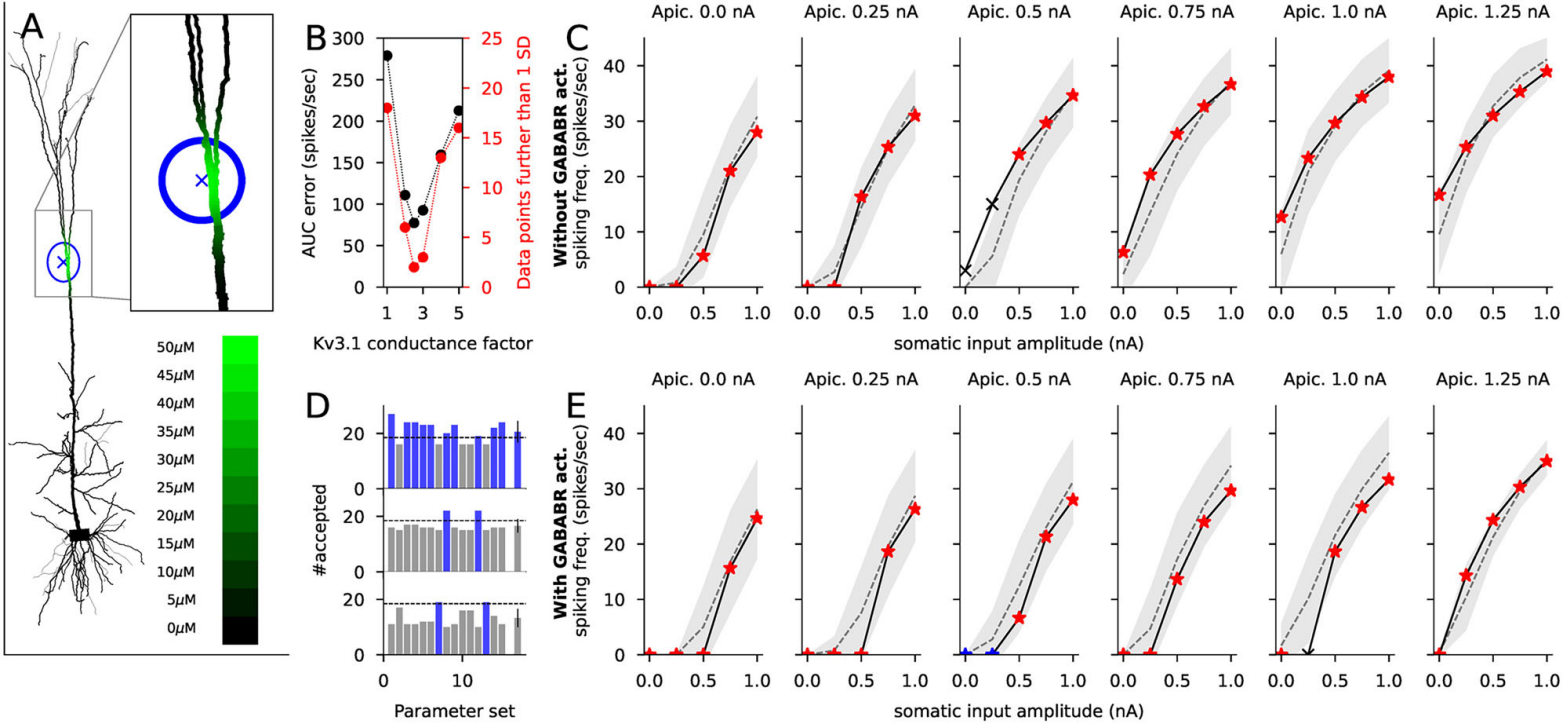
The model reproduces the GABA_B_R activation-dependent inhibition of the spiking response to apical and axo-somatic stimulation in L5PCs. ***A***, The morphology of the L5PC model (from Hay et al., 2011; apical dendrite extending upwards from the soma, basal dendrites to other directions) and the GABA_B_R agonist concentration at different compartments (color-coded). The locus of the simulated GABA_B_R agonist puff was at the main branching point of the apical dendrite (650 μm up from the soma), the maximal agonist concentration was 50 μM, and the SD of the agonist concentration was 45 μm. ***B***, The fitting of Kv3.1 conductance. The black data (left-hand side *y*-axis) shows the summed error of firing rate in response to a combination of axo-somatic [0.0, 0.25, 0.5, 0.75, or 1.0 nA; see (***C***)] and apical dendritic [0.0, 0.25, 0.5, 0.75, 1.0, or 1.25 nA; see (***C***)] DC stimulus between the predicted and experimentally measured (Schulz et al., 2021) data given different scaling factors (*x*-axis) of the Kv3.1 conductance in the absence of GABA_B_R activation. The red data (right-hand side *y*-axis) show the number of predicted data points that were further than 1 SD from the measured data. A scaling factor of 2.5 gave the smallest difference between model prediction and experimental data both in terms of total error (black) and number of correctly predicted data points (red) and was thus used henceforth. ***C***, The predicted (black curve with markers) and experimentally measured (gray curve with shaded area showing the mean ± SD) firing rates in response to the combined axo-somatic and apical dendritic stimulation in the absence of GABA_B_R activation. The *x*-axes show the amplitude of the axo-somatic DC stimulus, while the amplitude of the apical dendritic DC stimulus (0.0–1.25 nA) is indicated above the plots. The red asterisks and the black crosses indicate the model predictions that were closer or further, respectively, than 1 SD from the experimentally measured firing rate (Schulz et al., 2021). The model correctly predicted the firing rate for 28/30 stimulus combinations. ***D***, The number of correctly (prediction within 1 SD from experimental data) predicted firing rates in the presence of GABA_B_R activation using reaction-rate parameter sets from 15 different GABA_B_R activation model fits (Table S1). The blue bars label the parameter sets that correctly predicted the firing rate in the presence of GABA_B_R activation for two thirds (19/28) or more of the stimulus combinations that yielded acceptable predictions in the absence of GABA_B_R activation in (***C***). By contrast, the gray bars indicate parameter sets that correctly predicted the firing rate in the presence of GABA_B_R activation for 18 or fewer stimulus combinations. The top panel shows the results when a 45 μm SD of GABA_B_R agonist distribution was used, yielding optimal fit to the data, while the middle and bottom panels show the results where a too small (20 μm) or too large (100 μm) SD was used. The rightmost bars show the mean and SD across the 15 parameter sets. ***E***, The predicted (black curve with markers) and experimentally measured (gray) firing rate in response to the combined stimulus in the presence of GABA_B_R activation; see (***C***) for the GABA_B_R-blocked case. The model correctly (within 1 SD) predicted the firing rate for 27 out of the 28 stimulus combinations for which the GABA_B_R-blocked model correctly predicted the firing response, see (***C***). The model also correctly predicted the firing rate for two stimulus combinations for which the GABA_B_R-blocked model failed to predict the correct firing response (blue asterisks).

The data from Isaacson et al. (1993) allowed fitting the parameters of the biochemically detailed model that govern the kinetics of the postsynaptic GIRK channel activation, but it remains to be shown that the model can correctly capture the effects of GABA_B_R activation on input integration and AP generation in cortical cells. Here, we used a previous estimate of the single-channel conductance of the GIRK channel (33 pS; Chen and Johnston, 2005) and data from Schulz et al. (2021) where the AP firing profiles, i.e., the spiking response of L5PC to a combination of somatic and apical dendritic stimuli (5×6 data points) was recorded in presence and absence of GABA_B_R activation. We then implemented our biochemically detailed model of GABA_B_R pathway activation (Table 3) in a multicompartmental model of an L5PC (Hay et al., 2011; illustrated in Fig. 3*A*) to compare the simulated effects of postsynaptic GABA_B_R activation with the experimentally measured ones.

We first noticed that the Hay model did not fit well with the experimental data collected in the absence of GABA_B_R activation (Schulz et al., 2021)—instead, the predicted firing frequencies were constantly lower than observed in the data. Preliminary analyses, where the conductance of a single type of ion channels was altered, showed that increase of the Kv3.1 channel conductance, but not other alterations of ion-channel conductances, changed the AP firing profile of the model L5PC toward the data measured from (non-GABA_B_R-activated) L5PCs in Schulz et al. (2021). Although the Kv3.1 channels are hyperpolarizing, they exhibit a rapid channel deactivation that promotes fast repetitive firing in most neuron types, and they are expressed in L5PCs (Ichinohe et al., 2004; Kaczmarek and Zhang, 2017). The best fit to the data from Schulz et al. (2021) was obtained by using the Hay model where the Kv3.1 channel conductance was 150% larger than in the standard model: with this factor 28/30 of the predicted firing frequencies were within 1 standard deviation (SD) from those measured in Schulz et al. (2021; Fig. 3*B–C*).

We next implemented the biochemically detailed model of GABA_B_R activation (Table 3) and its coupling to a GIRK channel model, adapted from Yim et al. (2015), in the L5PC model. We repeated the simulations of combined somatic and apical dendritic stimuli in the presence of GABA_B_R activation using the 15 optimized biochemical models. In Schulz et al. (2021), it was estimated that the GABA_B_R agonist (50 μM) was effective up to a distance of 100 μm from the injection point at the apical dendrite 500–800 μm from the soma. To model this, we used a Gaussian density function centered at 650 μm toward the apical dendrite from the soma—the GABA_B_R agonist concentration was set 50 μM at the center and decreased with an SD of 20, 45, or 100 μm (Fig. 3*A*). It should be noted that we did not take into account the possible contribution of dendritic VGCCs to this inhibition—see Discussion. We found that the simulations performed with GABA_B_R agonist distribution with a SD of 45 μm fit well to the spiking data from Schulz et al. (2021): 29 of 30 somatic-apical stimulus combinations (including 27 of the 28 that were correctly reproduced in the absence of GABA_B_R activation) were correctly reproduced, i.e., such that the predicted value was within 1 SD from the value measured in Schulz et al. (2021), in the presence of GABA_B_R activation (Fig. 3*D–E*). This was a relatively robust result, since for 10/15 optimized parameter sets, two thirds (19/28) or more of the somatic-apical stimulus combinations were correctly reproduced in the presence of GABA_B_R activation.

Taken together, our model fits postsynaptic spiking data from L5PCs measured in the presence of GABA_B_R agonist well. Next, we use our model to explore the pre- and postsynaptic effects of GABA_B_R activation in L5PCs where activation is entirely driven by synaptic inputs.

### The effects of pre- and postsynaptic GABA_B_R activation on L5PC firing induced by in vivo-like synaptic inputs

Our common framework for modeling pre- and postsynaptic GABA_B_R activation offers a unique opportunity to compare the effects of the two. Here, we modeled the effects of both pre- and postsynaptic GABA_B_R stimulation on dendritic membrane potential and spiking activity during glutamatergically driven activity in an L5PC. To do this, we fit our model to in vivo patch-clamp-recorded data from L5PCs during electrical paw stimulation (Palmer et al., 2012). In Palmer et al. (2012), short electric pulses administered at the rat paw induced a dense spiking activity in L5PCs for 0.6 s. This activity was significantly weakened by GABA_B_R activation mediated by interhemispheric inputs (Palmer et al., 2012). We fit the model parameters pertaining to L5PC activity induced by this stimulation protocol (number of excitatory synapses and GABA flux at dendritic compartments) and used the obtained model for exploring the contributions of pre- and postsynaptic GABA_B_R activity to L5PC firing. Note that we here did not model the effects of GABA_B_R activation on postsynaptic voltage-gated Ca^2+^ channels although they, according to the results of Palmer et al. (2012), mediate part of the GABA_B_R-driven interhemispheric inhibition of L5PCs.

The L5PC postsynaptic response evoked by paw activation in Palmer et al. (2012) had a dynamic structure, where peaks and troughs of inputs alternated approximately every 50 ms for a total time of 600 ms. To model this, we stimulated 20 independent axons, modeled as in Figure 2, every 50 ms 12 times with a probability of 0.5 (i.e., each axon fired on average six times). A number (*N*_syn_) of synapses using the neurotransmitter-gated AMPA/NMDA model (see Methods) were distributed across the distal half of the apical dendritic tree of the L5PC (distance from soma >650 μm, longest branch length being 1,300 μm), and each was coupled to the neurotransmitter release model corresponding to one of the 20 (randomly chosen) presynaptic axon terminals. We repeated this stimulation 10 times with an interval of 10 s which was adequate for the L5PC to return to baseline. We added a stimulating somatic current lasting throughout the 600-ms periods whose amplitude was randomly picked (independently for each trial) from a uniform distribution between 0.15 and 0.25 nA—this produced a trial-to-trial variability of neuronal spike output, similar to Palmer et al. (2012). To model the interhemispheric inhibition of Palmer et al. (2012), we inserted GABA_B_Rs and GIRK channels activated by them to the distal half of the apical dendritic tree.

**Figure 4.**
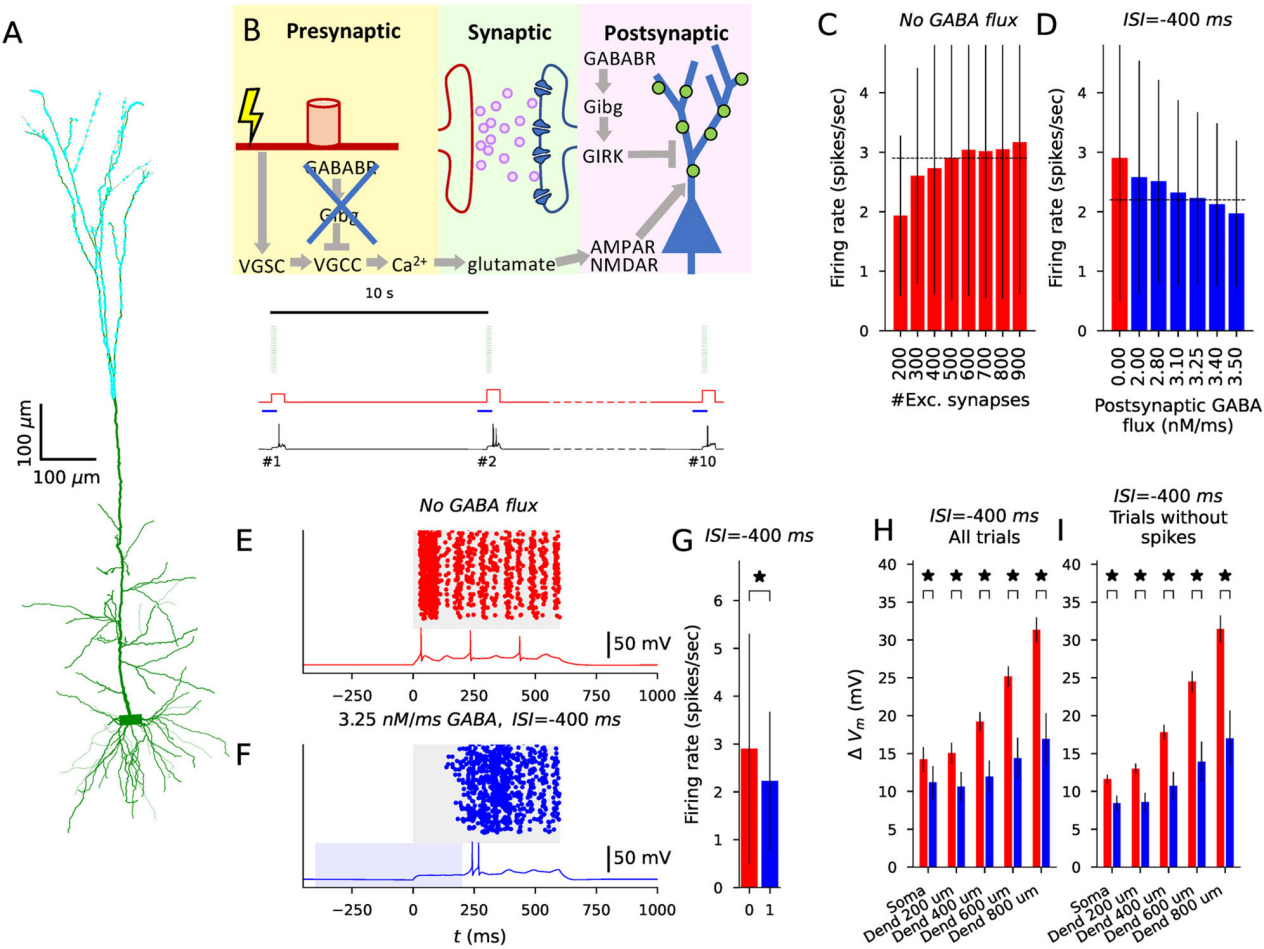
The model can be fitted to in vivo data on interhemispheric inhibition to explore the GABA_B_R-mediated suppression of high firing activity in L5PCs. ***A***, Illustration of the dendritic tree of the modeled L5PC (Hay et al., 2011; green) and the glutamatergic inputs (*N* = 500) distributed across the distal half of the apical dendrite (cyan). ***B***, Illustration of the GABA_B_R actions and the stimulation. Top: Presynaptic stimulation leads to activation of axonal voltage-gated Na^+^ channels (VGSCs), which opens the VGCCs, leading to glutamate release. The glutamate activates postsynaptic AMPARs and NMDARs, leading to depolarization of the membrane. When postsynaptic GABA_B_Rs are activated, the GIRK channels counteract this by hyperpolarizing the membrane. Here, the presynaptic GABA_B_Rs are inactive (indicated by the blue “X”) and all presynaptic VGCCs are in the “willing” state. Bottom: Illustration of the stimulation of the 20 axons (green), the concurrent subthreshold stimulation of postsynaptic L5PC soma (red), the periods of GABA_B_R activation (blue), and the resulting somatic membrane potential trace (black). ***C***, The firing rate (spikes/s) following the stimulus onset in the absence of postsynaptic GABA_B_R-activating inputs given different numbers of glutamatergic synaptic inputs. The stimulus consisted of 600 ms of rhythmic, 20 Hz stimulation of the axons where the probability of each stimulus was 0.5. The high-frequency stimulation (HFS) was combined with a synchronous and equally long (600 ms) DC at the soma with an amplitude randomly picked from a uniform distribution between 0.15 and 0.25 nA. We simulated L5PCs with 40 different randomly picked distributions of synapses, and each L5PC was given 10 independent stimuli administered every 10 s (i.e., each simulation lasted 100 s, plus a 10.6 s period for reaching a steady state and recording the output), leading to 400 trials. The data of Palmer et al. (2012; dashed line) was best reproduced by using *N* = 500 synapses. ***D***, The firing rate (spikes/s) following the stimulus onset in the presence of postsynaptic GABA_B_R-activating inputs given different fluxes of GABA. The GABA_B_R model (Table 3) was included in each compartment in the distal half of the L5PC. The onset of the GABA input was 400 ms before that of the glutamatergic inputs (ISI =−400 ms) and lasted for 600 ms. The data of Palmer et al. (2012) under interhemispheric inhibition (dashed line) was best reproduced by using the GABA flux of 3.25 nM/ms. ***E–F***, Illustration of the L5PC spiking across all trials and membrane potential time course of a single trial during the high-activity period (starting at *t* = 0 ms) in the absence (***E***) and presence (***F***) of the GABA flux of 3.25 nM/ms. The blue shading in (***F***) represents the timing of the GABA flux. ***G***, The L5PC firing rate during the high-activity period was significantly lower in the presence (blue; 3.25 nM/ms) than in the absence of the postsynaptic GABA_B_R-activating inputs (*U*-test, *p* = 0.022 < 0.05). ***H–I***, The average membrane potential during the high-activity period in the absence (red) and presence (blue) of the postsynaptic GABA_B_R-activating inputs. The asterisks denote statistical significance between absence and presence of postsynaptic GABA_B_R-activating inputs (*p* < 0.005) when averaged across all trials (***H***) or the trials where no APs were induced (***I***).

We first adjusted the amount of excitation in a single L5PC (number of glutamatergic synapses, Fig. 4*A*) in the absence of GABA_B_R activation (Fig. 4*B*) to fit to the firing rate (2.9 spikes/s) during the high-activity period. The best fit was obtained using 500 glutamatergic synapses (Fig. 4*C*)—i.e., each axon was connected to 25 synapses randomly placed on the distal apical dendrite. We then fit the amount of GABA flux (lasting 600 ms, starting 400 ms prior to the glutamatergic input) to the GABA_B_Rs to reproduce the lower firing frequency (2.2 spikes/s) in the presence of GABA_B_R activation. Following the observations of Palmer et al. (2012), we first excluded the GABA_B_R activation from the presynaptic terminals. The data from Palmer et al. (2012) was reproduced with a GABA flux of 3.25 nM/ms (Fig. 4*D*). The spiking patterns in the presence and absence of postsynaptic GABA_B_R activation are illustrated in Figure 4*E–F*, with the blue shading indicating the timing of the GABA_B_R activation.

Next, we tested whether the difference in the numbers of APs and dendritic membrane potentials between the absence and presence of GABA_B_R activation was significant. Similar to Palmer et al. (2012), the firing in the presence of GABA_B_R activation was significantly decreased compared to the absence (*U*-test, *p* = 0.02 < 0.05; *N* = 400, 400; Fig. 4*G*). However, contrary to Palmer et al. (2012), the average membrane potential during the 600 ms following the simulated paw stimulation was also significantly decreased in the presence of GABA_B_R activation compared to the absence (Fig. 4*H–I*). This was the case both when considering all trials (Fig. 4*H*) and when considering only the non-spiking trials (Fig. 4*I*), and the result held both for somatic membrane potentials as well as membrane potentials recorded at four different locations along the apical dendrite [*U*-test, *p* < 0.005, i.e., 0.05 Bonferroni-corrected by multiple (10) testings].

**Figure 5.**
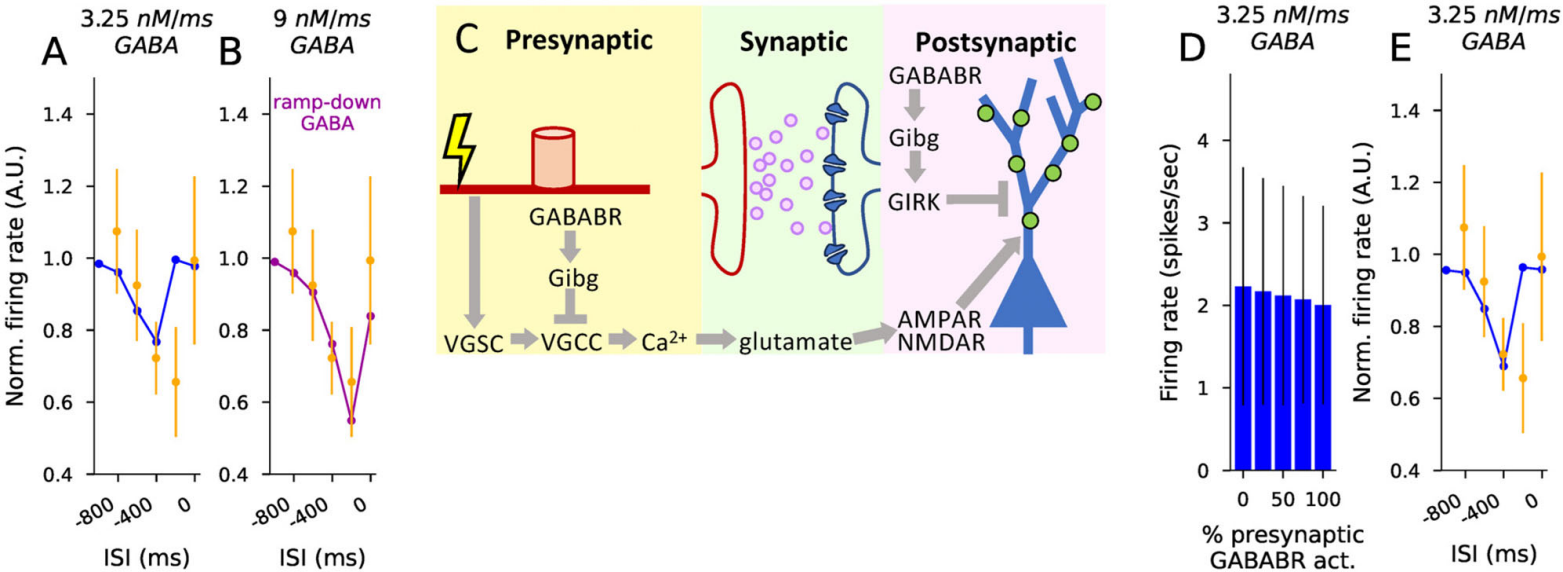
Presynaptic effects of GABA_B_R activation on interhemispheric inhibition are mild whereas the shape of the GABA_B_R activation significantly affects the sensitivity of the high firing activity to the onset of inhibition. ***A***, The normalized firing rate following the stimulus onset in the presence of postsynaptic GABA_B_R-activating inputs with varied time of onset. The firing rates were normalized by the corresponding firing rate in the absence of GABA_B_R-activating inputs. The orange dots show the data measured in Palmer et al. (2012), while the blue curves show the model predictions. ***B***, The normalized firing rate following the stimulus onset in the presence of a ramp-down form of postsynaptic GABA_B_R-activating inputs with varied time of onset. See Figure S2 for details. ***C***, Illustration of the stimulation and the GABA_B_R actions. In panels (***D***)–(***E***), the presynaptic GABA_B_Rs were activated in all or some of the 20 axons in addition to the postsynaptic GABA_B_R actions. ***D***, The firing rate following the stimulus onset in the presence of pre- and postsynaptic GABA_B_R-activating inputs. In these simulations, the presynaptic terminal was given the same GABA_B_R-activating inputs as the postsynaptic GABA_B_R-containing dendritic compartments, leading to the inactivation of some of the presynaptic N-type Ca^2+^ channels as in Figure 2. The fraction of presynaptic terminals (randomly picked among the 20) to which GABA_B_R-activating ligands were added is varied from 0% (the data from the blue bar of Fig. 4*G*) to 100% (all presynaptic terminals were given GABA inputs). ***E***, The experiments of panel (***A***) repeated by administering both pre- and postsynaptic GABA_B_R-activating inputs as in (***D***).

We also tested the effect of the timing of the GABA_B_R activation on the L5PC firing during the high-activity period. As observed in Palmer et al. (2012), our model predicts that GABA_B_R activity that is either synchronous with the glutamatergic activity or preceding it by too much (GABA_B_R activity onset ≥800 ms before the onset of the glutamatergic inputs) does not suppress the L5PC firing during the high-activity period (Fig. 5*A*). For an interval of 200 ms, however, our model predicted normal firing rate while the data of Palmer et al. (2012) suggested a strong suppression of L5PC firing during the high-activity period. By contrast, a strong suppression for the 200 ms interval was obtained if we modified the simulation to use a ramp-down GABA stimulus instead of the square-pulse GABA stimulus of Figures 4*D–I* and 5*A* (Figs. 5*B* and S2).

We next explored the role of presynaptic GABA_B_R activation in inhibiting L5PC activity (Fig. 5*C*). First, we carried out simulations where the presynaptic VGCCs were externally set to be maximally G_i*βγ*_-bound regardless of the GABA inputs, and the postsynaptic GABA_B_R activation was the same as before (a square-pulse flux of 3.25 nM/ms for 600 ms, starting 400 ms before the glutamatergic stimulation). If we assumed all VGCCs to be G_i*βγ*_-bound, we achieved a firing rate of 1.58 spikes/s (difference to postsynaptic-only GABA_B_R activation was Δ*f* = −0.65 spikes/s) during the high-activity period, whereas if we chose a quasi-maximal level of G_i*βγ*_-bound VGCCs, that is, a steady state implied by the assumption of all G_i_ being dissociated and the ratio of the forward and backward rates of G_i*βγ*_ binding to VGCC (which resulted in 85.7% of VGCCs being G_i*βγ*_-bound), we achieved a firing rate of 1.75 spikes/s (Δ*f* = −0.48 spikes/s; Fig. S3*A*). The effects of the presynaptic VGCC inhibition were not linearly cumulative with those of the postsynaptic GABA_B_R activation: when blocking the postsynaptic GABA_B_R activity, the inclusion of VGCC inhibition decreased the firing rate from 2.9 spikes/s only to 2.66 spikes/s ( $\Delta f_{\mathrm{no}\;\mathrm{postsyn}.\;\mathrm{GAB}A_{B}R}=-0.24$ spikes/s) or 2.71 spikes/s ( $\Delta f_{\mathrm{no}\;\mathrm{postsyn}.\;\mathrm{GAB}A_{B}R}=-0.19$ spikes/s) in the maximal and quasi-maximal modes of VGCC inhibition, respectively (Fig. S3*A*).

Finally, we tested whether similarly large effects were obtained by applying presynaptic GABA inputs similar to those affecting the postsynaptic GABA_B_Rs (a square-pulse flux of 3.25 nM/ms for 600 ms, starting 400 ms before the glutamatergic stimulation). Depending on the fraction of presynaptic terminals affected by GABA, the firing rate of the L5PC was 2.0–2.17 spikes/s (Δ*f* = −0.06–0.23 spikes/s; Fig. 5*D*). These changes were statistically non-significant, concordant with the observations of Palmer et al. (2012). The presence of presynaptic GABA_B_R activation also had little effect on the sensitivity of the L5PC firing to the timing of the GABA_B_R-activating stimulus (Fig. 5*E*). We also tested if presynaptic GABA_B_R activation could have larger effects on the L5PC firing if its internal mechanisms and the GABA binding were enhanced. To do this, we increased the GABA flux and decreased the RGS concentration in the presynaptic terminals. However, these changes had mild strengthening effects of the GABA_B_R-mediated inhibition of the L5PC firing: either increasing the presynaptic GABA flux twofold to fourfold (Fig. S3*B*) or decreasing the presynaptic RGS concentration by 25–90% (Fig. S3*C*) only decreased the firing during the high-activity period to 1.96–2.0 spikes/s (Δ*f* = 0.23 − 0.27 spikes/s).

Taken together, the predictions obtained with our model fitted to experimentally measured features of interhemispheric inhibition highlight the role of postsynaptic rather than presynaptic GABA_B_R activation and suggest that the GABA input activating the postsynaptic GABA_B_Rs is strong shortly after the onset of the ipsilateral stimulation and weaker afterwards. While our model predicts that by completely inhibiting presynaptic VGCCs can significantly add to the interhemispheric inhibition mediated by postsynaptic GABA_B_R activation, it also suggests that such a degree of VGCC inhibition cannot be achieved by presynaptic GABA_B_R activation alone.

### Model-aided analysis of RGS effects on neuronal dynamics

The biochemically detailed nature of our model permits using it for explaining and studying the implications of data from genetic knockout experiments. In addition to the proteins that directly mediate the effects of GABA_B_R activation, such as the GIRK channels, VGCCs, and the GABA_B_Rs themselves, an important class of proteins included in our model are the RGS proteins. A primary role of RGS proteins, especially the R7 family (RGS6, RGS7, RGS9, and RGS11), is to terminate the G-protein activity by facilitating the GTPase activity of the G*α* subunit, but they also mediate other G-protein-related actions through forming a complex with other proteins (Anderson et al., 2009). In a genetic knockout experiment (Ostrovskaya et al., 2014), the authors showed that RGS7 contributed to termination of GABA_B_R-mediated postsynaptic GIRK currents in CA1 pyramidal neurons, but they also reported an RGS7-mediated increase in the sensitivity of GIRK channels to GABA_B_R activation. Namely, RGS7-KO neurons responded to approximately five times smaller GABA_B_R agonist concentrations than wild-type neurons (Ostrovskaya et al., 2014). Importantly, the authors also reported that the RGS7-KO altered the CA1 neuron excitability and the amplitude of low-frequency stimulation (LFS) induced long-term depression (LTD; Ostrovskaya et al., 2014). The observed effects could have been a direct consequence of the decreased amount of RGS proteins, but they could also stem from increased sensitivity of GIRK channels to GABA_B_R activation. While the prolonged termination of GIRK currents is likely to be a direct consequence of the former, it remains unknown whether changes in firing threshold and plasticity are created by the former or the latter cause. Here, we showcase the potential of our model in answering mechanistic questions such as this one by analyzing the effects of different manipulations of the GABA_B_R system in a multicompartmental CA1 pyramidal neuron model (Combe et al., 2018).

**Figure 6.**
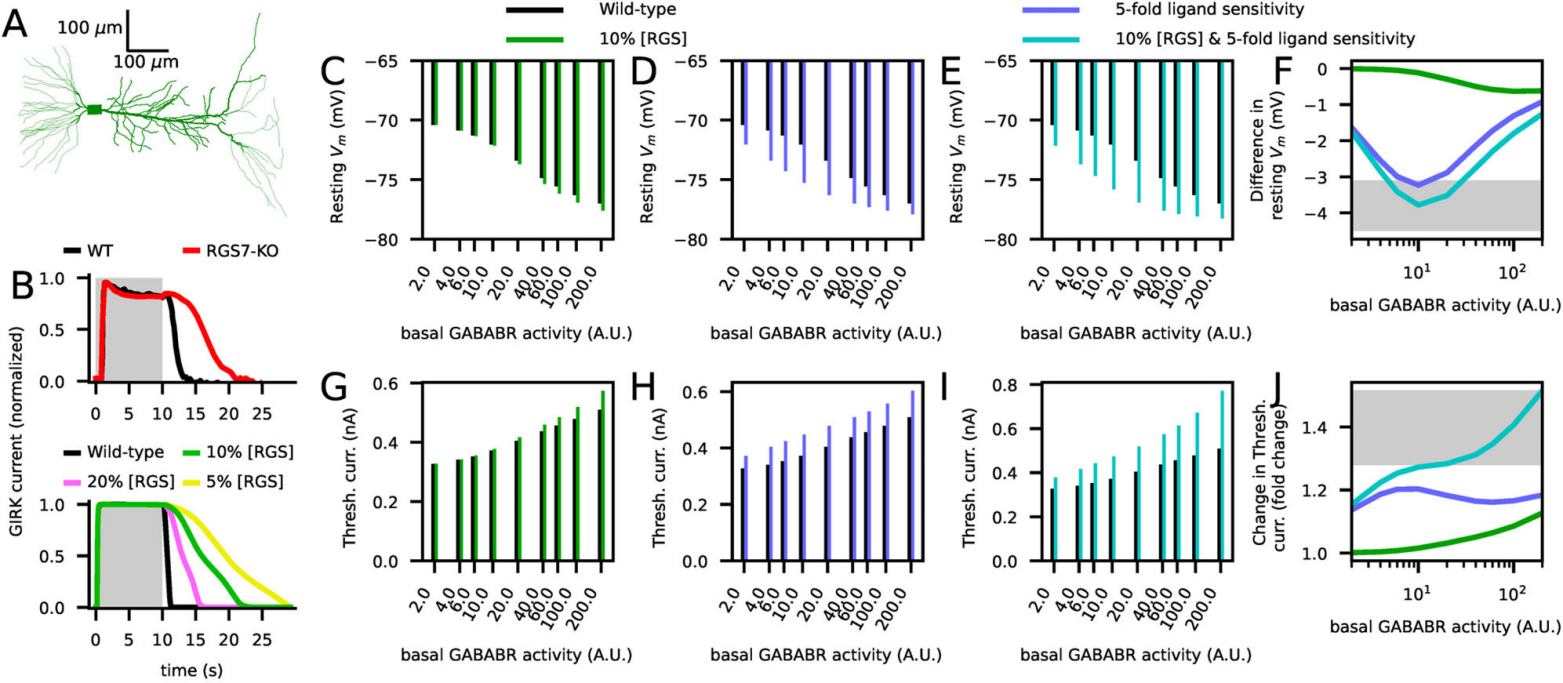
Reduction of RGS concentration to 10% and fivefold sensitivity to GABA_B_R agonists prolongs GABA_B_R-mediated GIRK currents, hyperpolarizes the membrane, and increases the firing threshold in CA1 pyramidal cells in accordance with experimental data from an RGS7-KO. ***A***, The morphology of the Combe CA1 neuron model (Combe et al., 2018). ***B***, Top panel: Time course of baclofen-activated GIRK currents in CA1 pyramidal cells of wild-type (black) and RGS7-KO (red) mice (data extracted from Ostrovskaya et al., 2014). Bottom panel: Model predictions for GIRK currents recorded at the apical dendritic compartment closest to soma in response to GABA_B_R activation by a saturating agonist concentration (100 μM) at the default (Table 3B) RGS concentration (black) and when the RGS concentration was reduced to 20% (pink), 10% (green), or 5% (yellow). The RGS reduction by 90% (green) fit well the experimental observations (top panel). The time of baclofen administration is indicated by the shaded areas. ***C–E***, Model predictions for somatic resting membrane potential in the Combe model integrated with the GABA_B_R model (i.e., the “wild-type” model) as a function of basal GABA_B_R activity (modeled using a constant, non-depleting GABA_B_R agonist concentration such that 1 A.U. corresponds to 1 pM agonist concentration). The black bars represent the Combe model integrated with the default GABA_B_R model (Table 3), whereas the colored bars represent the candidate models for RGS7-KO; namely, the GABA_B_R model where the RGS concentration was reduced by 90% (green; ***C***), where the sensitivity to GABA_B_R agonist was fivefold larger (blue; ***D***), or where both manipulations were present (cyan; ***E***). ***F***, The difference between the resting membrane potential in the wild-type model and the candidate models for RGS7-KO as a function of basal GABA_B_R activity. The shaded area represents the mean ± SD of the experimentally observed difference in resting membrane potentials between wild-type and RGS7-KO mice (Ostrovskaya et al., 2014). ***G–I***, Model predictions for the threshold current (for a somatic square-pulse current of 5 ms) in the wild-type model (black) and the candidate models of RGS7-KO [green, blue, cyan—see (***C***)–(***E***)]. ***J***, The ratio of threshold current in the wild-type model to that in the candidate models of RGS7-KO as a function of basal GABA_B_R activity. The shaded area represents the mean ± SD of the experimentally observed ratio of threshold currents between wild-type and RGS7-KO mice (Ostrovskaya et al., 2014).

We tested the effects of partial removal of RGS and the altered baclofen (GABA_B_R agonist) sensitivity on the GIRK current deactivation in CA1 pyramidal cells (Fig. 6*A*). This was done in the absence of synaptic stimulation. A 90% reduction in RGS concentration caused the same magnitude of prolongation of the GIRK current activity (≈ 10 s) as observed in RGS7-KO (Ostrovskaya et al., 2014; Fig. 6*B*), suggesting a minor contribution of RGS protein other than RGS7 to GIRK current termination in CA1 pyramidal cells. However, 90% RGS reduction alone decreased the somatic resting membrane only by 0.01–0.6 mV depending on the basal level of GABA_B_R activity (Fig. 6*C*). By contrast, a fivefold increase in GABA_B_R agonist sensitivity (simulated by using fivefold larger concentration) decreased the resting membrane by 1.0–3.2 or 1.3–3.8 mV (depending on the basal level of GABA_B_R activity) when unaccompanied or accompanied, respectively, by the 90% RGS reduction (Fig. 6*D–E*). Likewise, the 90% RGS reduction alone had little effect on the firing threshold in response to somatic stimulus (Fig. 6*G*), but a fivefold increase in GABA_B_R agonist sensitivity, with (Fig. 6*H*) or without (Fig. 6*I*) the reduction in RGS concentration, increased the firing threshold by 14–18% or 15–51%, respectively, depending on the basal level of GABA_B_R activity. The experimentally measured effects of RGS7-KO on resting membrane potential (−3.8 mV ± 0.7 mV; Fig. 6*F*) and threshold current (140% ± 12%; Fig. 6*J*) in CA1 pyramidal neurons were best approximated by our model with the 90% RGS reduction and a fivefold increase in GABA_B_R agonist sensitivity given a basal GABA_B_R activity of approximately 10 A.U. (Fig. 6*F,J*; cyan).

**Figure 7.**
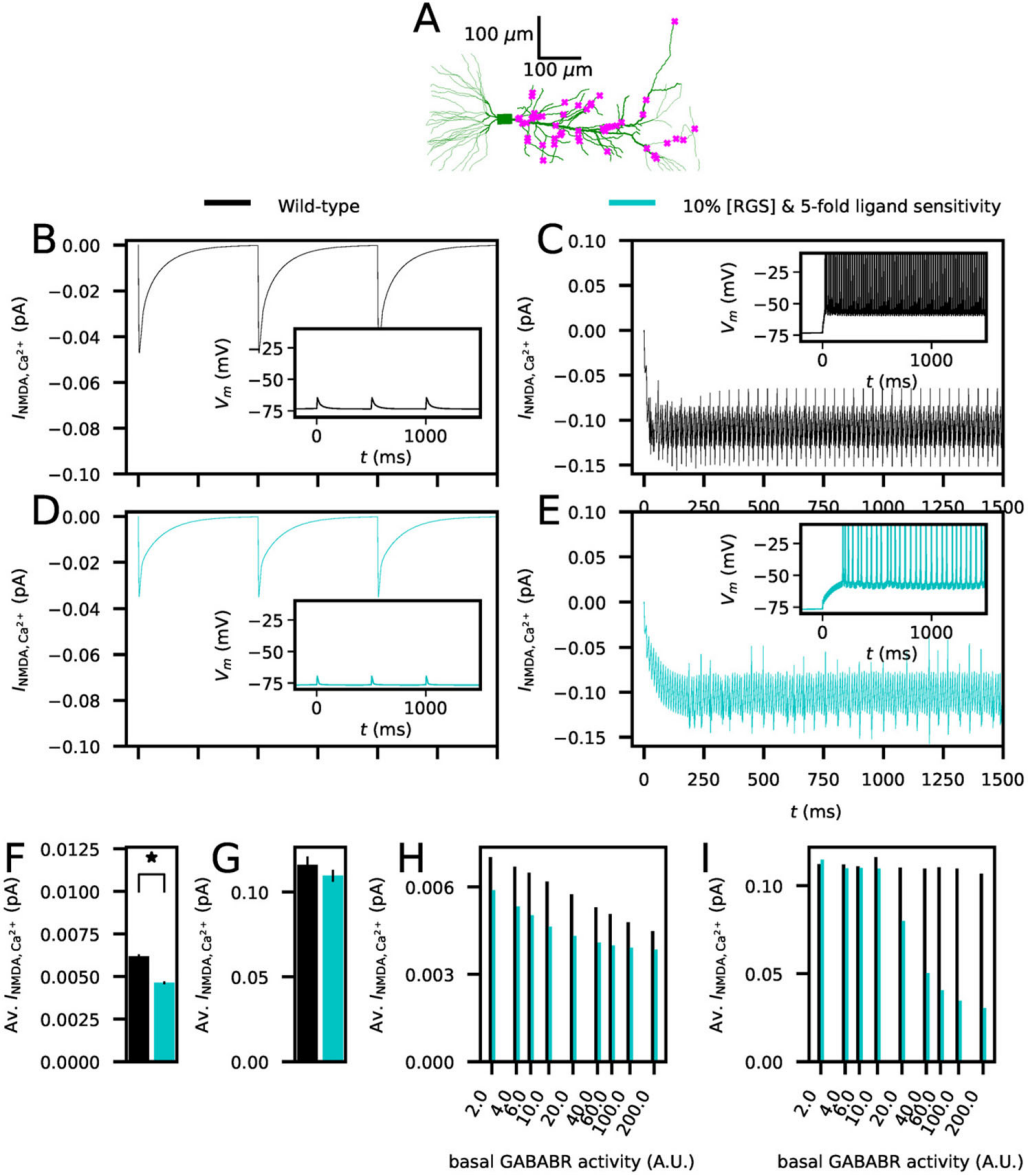
Reduction of RGS concentration to 10% and fivefold sensitivity to GABA_B_R agonists decreases the Ca^2+^ transients induced by LFS in CA1 pyramidal cells as suggested by experimental data from an RGS7-KO. ***A***, The morphology of the Combe CA1 neuron model (Combe et al., 2018; green) and an illustration of the 50 synapses randomly placed at the apical dendrite (magenta). ***B–C***, NMDAR-mediated Ca^2+^ currents (averaged over the 50 synapses distributed across the apical dendrite) in response to the 2 Hz (***B***) or 100 Hz (***C***) stimulation according to the wild-type model. The basal GABA_B_R activity was 10 A.U. (Fig. 6). Insets: Somatic membrane potential time course. ***D–E***, Simulations of (***B***)–(***C***) repeated for the candidate RGS7-KO model where RGS concentration was reduced by 90% and the sensitivity to GABA_B_R agonist was fivefold larger. ***F–G***, The predicted NMDAR-mediated Ca^2+^ currents in the 2 Hz (***F***) and 100 Hz (***G***) protocol. The NMDAR-mediated Ca^2+^ currents were first averaged across the stimulus interval (500 or 10 ms), and the mean across the 10 last stimuli were calculated. The bars show the mean and SD of *N* = 5 simulations. In the 2 Hz protocol, the Ca^2+^ current amplitudes were significantly smaller (*U*-test, *p* = 0.009 < 0.05) in the RGS7-KO model compared to the wild-type model in response to 2 Hz stimulus, but the difference in the Ca^2+^ current amplitudes in response to the 100 Hz stimulus did not reach significance (*U*-test, *p* = 0.076 > 0.05). ***H–I***, The mean NMDAR-mediated Ca^2+^ current amplitudes in the 2 Hz (***H***) and 100 Hz (***I***) protocol for the wild-type and RGS7-KO model across different basal GABA_B_R activity levels. The RGS7-KO model predicts a difference in the HFS protocol only for large basal GABA_B_R activity, whereas for the LFS protocol the Ca^2+^ current amplitudes are lower in the RGS7-KO model throughout the tested GABA_B_R activity levels.

We next simulated the effect of RGS7-KO on Ca^2+^ inputs during stimulation protocols that induced plasticity in CA3–CA1 synapses. To do this, we distributed 50 AMPA/NMDAR synapses (Wang, 1999; Mäki-Marttunen and Mäki-Marttunen, 2022) randomly across the apical dendrite of the CA1 pyramidal neuron model (Fig. 7*A*) and stimulated them rhythmically either at a 2 Hz (LFS) or 100 Hz (HFS). The NMDAR-mediated Ca^2+^ currents were significantly larger in HFS stimulation compared to LFS stimulation (Fig. 7*B–I*), owing to the induction of spiking in response to the HFS but not LFS protocol according to our model (Fig. 7*B–C*, insets). Our model of RGS7-KO (90% RGS reduction in RGS concentration and fivefold increase in GABA_B_R agonist sensitivity) in moderate basal GABA_B_R activity (10 A.U.) expressed significantly smaller NMDAR-mediated Ca^2+^ currents in response to LFS (Fig. 7*B,D,F*) but unaltered NMDAR-mediated Ca^2+^ currents in response to HFS (Fig. 7*C,E,G*), which is in accordance with the observations of decreased amplitude of LFS-induced LTD and unaltered HFS-induced long-term potentiation (LTP; Ostrovskaya et al., 2014). However, our model suggested that in higher levels of basal GABA_B_R activity the Ca^2+^ currents are decreased in response to both LFS (Fig. 7*H*) and HFS (Fig. 7*I*) in RGS7-KO.

Taken together, our model suggests that both a 90% reduction in RGS concentration and a fivefold increase in GABA_B_R agonist sensitivity are required for the effects of RGS7-KO on the GIRK current deactivation time, resting membrane potential and firing threshold in CA1 pyramidal cells. Our model also suggests that the difference between experimentally observed effects of RGS7-KO on LFS-induced LTD compared to HFS-induced LTP is due to the RGS7-KO significantly decreasing the NMDAR-mediated Ca^2+^ current amplitudes in response to LFS but not those in response to HFS.

## Discussion

Here, we developed a biochemically detailed model of GABA_B_Rs and their effects on neuronal excitability through their G_i*βγ*_-mediated interactions with GIRK channels and VGCCs. Our model has two important advances compared to existing models. Firstly, the model describes the GABA_B_R and G_i*βγ*_ activity in both pre- and postsynaptic terminals. The reaction rates were the same in different domains, only the concentrations of proteins (here, the RGS proteins) varied depending on the cell type and location. Thanks to this design principle, the model can be flexibly used in other cell types as well—only the concentrations of the proteins involved may need to be adjusted to brain-area-specific data. Secondly, we described the mass-action-law-based interactions between GABA_B_Rs, G_i_ protein subunits, RGS proteins, and the target proteins (GIRK channels and VGCCs) instead of relying on simplified dynamical interdependencies. Including these interactions permits integration with larger models (e.g., models describing the effects of G_i*α*_ activation) and testing hypotheses on the effects of genetic manipulations of the involved proteins as well as pharmacological manipulations. In comparison, most models of GABA_B_R-mediated K^+^ conductance treat it essentially as a postsynaptic conductance with predefined time course (Traub et al., 1994; Turi et al., 2019). Of the previous biochemically detailed models, the model of Destexhe and Sejnowski (1995) describes the dynamics of the postsynaptic GABA_B_Rs and G-proteins (and how they activate K^+^ currents) in a simplified manner (no separate *α* or *β*–*γ* subunits and no binding to GIRK channels). In Li et al. (2020), GABA activates presynaptic GABA_B_Rs and mediates short-term depression, but no G-protein interactions are described. To our knowledge, our model is the first to incorporate detailed data on the dependence of GIRK channel conductance (Dascal and Kahanovitch, 2015) and VGCC voltage-dependence and kinetics (Huynh et al., 2015) on G_i*βγ*_ binding into the GABA_B_R model.

We applied our model to data from RGS7-KO (Ostrovskaya et al., 2014) to show the potential of our model in complementing experimental data to explain the mechanisms of genetic manipulations. In Ostrovskaya et al. (2014), two key effects of RGS7-KO were reported: the decay time of GIRK conductance after a period of saturating level of the GABA_B_R agonist baclofen was significantly extended, and the sensitivity to baclofen (in the domain of non-saturating concentrations) was increased fivefold. While the first effect is likely to be a direct effect of decreased concentration of RGS proteins, the latter effect may be a subtype-specific interaction (Ostrovskaya et al., 2014) or another, yet unknown mechanism. Here, we applied a model where we used five times larger GABA_B_R agonist concentration to effectively capture the increased baclofen-sensitivity of RGS7-KO reported in Ostrovskaya et al. (2014). We showed that both of these subcellular-level effects (decreased concentration of the G_i*α*_-GTP-hydrolyzing RGS proteins and increased sensitivity to GABA_B_R ligands) were needed to explain the electrophysiological phenotypes of the RGS7-KO, namely, the decreased resting membrane potential and the increased firing threshold. We also found out that the effects of RGS7-KO on plasticity in CA1 pyramidal cells (decreased LTD amplitude but unaffected LTP amplitude Ostrovskaya et al., 2014) are likely to be a direct consequence of the stimulus-induced postsynaptic Ca^2+^ flux being significantly reduced in the RGS7-KO model during LFS but not HFS.

The level of biochemical detail in our model makes it a strong candidate for dissecting the effects of GABA_B_R activation on brain activity at multiple levels. Specifically, only neuron models in which the effects of GABA_B_R activation are constrained by physiological limits—e.g., the strength of GABABR-mediated hyperpolarization at the postsynaptic dendrite should not exceed the limits imposed by the density and single-channel conductance of the GIRK channels—allow computational modeling to extract reliable information about the processes underlying the observed neuronal phenomena. In this work, we used our model to explore the neural mechanisms of interhemispheric inhibition utilizing data from Palmer et al. (2012; Fig. 4). Most of the characteristics of the firing in response to contralateral paw stimulation and its GABA_B_R-mediated inhibition by ipsilateral paw stimulation were reproduced in our single-L5PC model. However, there was a mismatch between the sensitivity of the GABA_B_R-mediated inhibition to the timing of the inputs (Fig. 5*A,E*). This mismatch could be due to the dynamic structure of the predicted L5PC firing pattern, which features an initial high-frequency firing phase (Fig. 4*E–F*), but it could also result from an unrealistic GABA input profile. Here, we explored the latter scenario, and found that our simulation results closely match experimental data on the timing sensitivity of GABA inputs when a ramp-down, rather than a square-pulse, form of GABA_B_R activation was used (Figs. 5*B* and S2). This suggests that GABA_B_R activation associated with interhemispheric inhibition may be strong shortly after the ipsilateral paw stimulation and weaker later on. Such a scenario could be mediated by short-term depression either in the input (Price et al., 2005) or output (Oláh et al., 2009; Chittajallu et al., 2013; Abs et al., 2018) of the neurogliaform neurons, which are the main mediators of postsynaptic GABA_B_R activation in the cortex (Oláh et al., 2009; Schulz et al., 2021). Their late spiking characteristics (Oláh et al., 2007) and the abovementioned observations of their synaptic depression make it plausible that the GABA signal activating the postsynaptic GABA_B_Rs was mediated by few APs in presynaptic neurogliaform neurons and had its strongest impact at the start of the spiking response of these neurons.

The impact of GABA_B_R activation at the network level is complex as it mediates postsynaptic GIRK channel activation and presynaptic short-term depression in both excitatory and inhibitory presynaptic terminals. Here, we modeled the effect of GABA_B_R activation in all three phenomena, and we also compared the effects of pre- and postsynaptic GABA_B_R activation on L5PC spiking under an in vivo-like synaptic stimulation. When implemented together with the vesicle release model as in the experiments of Figures 2 and 4, the model allows the analysis of how two types of presynaptic short-term depression, namely, the one mediated by GABA_B_R activation and VGCC inactivation and the one mediated by depletion of readily releasable neurotransmitter vesicles, interact and counteract. This, together with future description of GABA_B_R-meditated effects on the vesicular release machinery (Manz et al., 2019) and alternative mechanisms of short-term plasticity [e.g., mediated by metabotropic glutamate receptors (Scanziani et al., 1997) or adenosine receptors (Brager and Thompson, 2003)], will allow a biophysically and biochemically detailed analysis that captures the most relevant molecular aspects of short-term dynamics in synapses.

GABA_B_R activation inhibits VGCCs also in the dendrites (Pérez-Garci et al., 2006, 2013; Palmer et al., 2012; Schulz et al., 2021). In this work, we only considered the presynaptic N-type Ca^2+^ channels, which display a strong shift in voltage-dependency in response to binding with G_i*βγ*_ (Colecraft et al., 2000), but notable effects have also been measured in P/Q-type channels (Huynh et al., 2015). Previous work on non-neuronal cells also shows the modulation of L-type channels by G_i*βγ*_ binding (Ivanina et al., 2000). The significance of GABA_B_R-mediated inhibition of L-type Ca^2+^ channels in modulating pyramidal neuron firing activity has been shown (Pérez-Garci et al., 2013; Schulz et al., 2021), and the reports of N- and P/Q-type Ca^2+^ channels being expressed at pyramidal cell dendrites (Lorenzon and Foehring, 1995; Almog and Korngreen, 2014) suggest that the total contribution of VGCCs to the postsynaptic effects of GABA_B_R activation may be substantial. In the modeling experiments of Figure 3, we assumed that all inhibition of the L5PC firing by GABA_B_R activation was mediated by GIRK channels. The experiments of Schulz et al. (2021) showed that there was a significant interaction between nimodipine (an L-type Ca^2+^ channel inhibitor) application and stimulus intensity in modulating the effect of baclofen on spiking rate but that the nimodipine did not have a significant main effect. Likewise, in the modeling experiments of Figure 4, we achieved a GABA_B_R-mediated decrease in L5PC firing that was mediated by GIRK channel activation only, although the experiments of Palmer et al. (2012) showed that some of the effects of baclofen on L5PC firing were mediated by VGCCs. Some of the estimates of the system-level parameters fitted in the experiments of Figures 3 and 4 should therefore be revised once a functional model taking into account the effects of GABA_B_R activation on dendritic VGCCs becomes available.

The GABA_B_R system is a prominent candidate for the development of treatments for mental disorders (Evenseth et al., 2020). Our model can be readily implemented in different brain regions, such as the striatum (Kupferschmidt and Lovinger, 2015), and used to study the effects of genetic disposition and pharmacological manipulations of the proteins involved in the GABA_B_R network. In addition to manipulations of postsynaptic GABA_B_R pathway proteins showcased here, our model can be used for biochemically detailed simulation of the constituents of presynaptic short-term depression, alterations of which have been postulated as a mechanism for addiction (Johnson and Lovinger, 2016). Computational analyses of relevant neuron populations by our model could thus reveal mechanisms of many mental disorders and suggest novel treatment options.

## References

[B1] Abs E, et al. (2018) Learning-related plasticity in dendrite-targeting layer 1 interneurons. Neuron 100:684–699. 10.1016/j.neuron.2018.09.001 30269988 PMC6226614

[B2] Almog M, Korngreen A (2014) A quantitative description of dendritic conductances and its application to dendritic excitation in layer 5 pyramidal neurons. J Neurosci 34:182–196. 10.1523/JNEUROSCI.2896-13.2014 24381280 PMC6608163

[B3] Anderson GR, Posokhova E, Martemyanov KA (2009) The R7 RGS protein family: multi-subunit regulators of neuronal G protein signaling. Cell Biochem Biophys 54:33–46. 10.1007/s12013-009-9052-9 19521673 PMC2827338

[B4] Bahl A, Stemmler MB, Herz AV, Roth A (2012) Automated optimization of a reduced layer 5 pyramidal cell model based on experimental data. J Neurosci Methods 210:22–34. 10.1016/j.jneumeth.2012.04.006 22524993

[B5] Bettler B, Kaupmann K, Mosbacher J, Gassmann M (2004) Molecular structure and physiological functions of GABAB receptors. Physiol Rev 84:835–867. 10.1152/physrev.00036.2003 15269338

[B6] Bianchi D, Marasco A, Limongiello A, Marchetti C, Marie H, Tirozzi B, Migliore M (2012) On the mechanisms underlying the depolarization block in the spiking dynamics of ca1 pyramidal neurons. J Comput Neurosci 33:207–225. 10.1007/s10827-012-0383-y 22310969

[B7] Brager DH, Thompson SM (2003) Activity-dependent release of adenosine contributes to short-term depression at CA3-CA1 synapses in rat hippocampus. J Neurophysiol 89:22–26. 10.1152/jn.00554.2002 12522156

[B8] Burnashev N, Zhou Z, Neher E, Sakmann B (1995) Fractional calcium currents through recombinant GluR channels of the NMDA, AMPA and kainate receptor subtypes. J Physiol 485:403–418. 10.1113/jphysiol.1995.sp020738 7666365 PMC1158001

[B9] Chen X, Johnston D (2005) Constitutively active G-protein-gated inwardly rectifying K+ channels in dendrites of hippocampal CA1 pyramidal neurons. J Neurosci 25:3787–3792. 10.1523/JNEUROSCI.5312-04.2005 15829630 PMC6724929

[B10] Chittajallu R, Pelkey KA, McBain CJ (2013) Neurogliaform cells dynamically regulate somatosensory integration via synapse-specific modulation. Nat Neurosci 16:13–15. 10.1038/nn.3284 23222912 PMC4132638

[B11] Colecraft HM, Patil PG, Yue DT (2000) Differential occurrence of reluctant openings in G-protein–inhibited N-and P/Q-type calcium channels. J Gen Physiol 115:175–192. 10.1085/jgp.115.2.175 10653895 PMC2217198

[B12] Combe CL, Canavier CC, Gasparini S (2018) Intrinsic mechanisms of frequency selectivity in the proximal dendrites of ca1 pyramidal neurons. J Neurosci 38:8110–8127. 10.1523/JNEUROSCI.0449-18.2018 30076213 PMC6146492

[B13] Craig MT, McBain CJ (2014) The emerging role of GABAB receptors as regulators of network dynamics: fast actions from a ‘slow’ receptor? Curr Opin Neurobiol 26:15–21. 10.1016/j.conb.2013.10.002 24650499 PMC4024344

[B14] Dascal N, Kahanovitch U (2015) The roles of G*βγ* and G*α* in gating and regulation of GIRK channels. Int Rev Neurobiol 123:27–85. 10.1016/bs.irn.2015.06.001 26422982

[B15] Deb K, Pratap A, Agarwal S, Meyarivan T (2002) A fast and elitist multiobjective genetic algorithm: NSGA-II. IEEE Trans Evol Comput 6:182–197. 10.1109/4235.996017

[B16] Destexhe A, Mainen ZF, Sejnowski TJ (1994) Synthesis of models for excitable membranes, synaptic transmission and neuromodulation using a common kinetic formalism. J Comput Neurosci 1:195–230. 10.1007/BF00961734 8792231

[B17] Destexhe A (1998) Spike-and-wave oscillations based on the properties of GABAB receptors. J Neurosci 18:9099–9111. 10.1523/JNEUROSCI.18-21-09099.1998 9787013 PMC6793559

[B18] Destexhe A, Sejnowski TJ (1995) G protein activation kinetics and spillover of gamma-aminobutyric acid may account for differences between inhibitory responses in the hippocampus and thalamus. Proc Natl Acad Sci 92:9515–9519. 10.1073/pnas.92.21.9515 7568165 PMC40832

[B19] Evenseth LSM, Gabrielsen M, Sylte I (2020) The GABAB receptor-structure, ligand binding and drug development. Molecules 25:3093. 10.3390/molecules25133093 32646032 PMC7411975

[B20] Ghit A, Assal D, Al-Shami AS, Hussein DEE (2021) GABAA receptors: structure, function, pharmacology, and related disorders. J Genet Eng Biotechnol 19:123. 10.1186/s43141-021-00224-0 34417930 PMC8380214

[B21] Groen MR (2011) Two modes of GABAB: specific localized inhibition and global network inhibition. J Neurosci 31:8327–8328. 10.1523/JNEUROSCI.1957-11.2011 21653837 PMC6623342

[B22] Hay E, Hill S, Schürmann F, Markram H, Segev I (2011) Models of neocortical layer 5b pyramidal cells capturing a wide range of dendritic and perisomatic active properties. PLoS Comput Biol 7:e1002107. 10.1371/journal.pcbi.1002107 21829333 PMC3145650

[B23] Huynh TG, Cuny H, Slesinger PA, Adams DJ (2015) Novel mechanism of voltage-gated N-type (Cav2.2) calcium channel inhibition revealed through *α*-conotoxin Vc1.1 activation of the GABAB receptor. Mol Pharmacol 87:240–250. 10.1124/mol.114.096156 25425625

[B24] Ichinohe N, Watakabe A, Miyashita T, Yamamori T, Hashikawa T, Rockland K (2004) A voltage-gated potassium channel, kv3. 1b, is expressed by a subpopulation of large pyramidal neurons in layer 5 of the macaque monkey cortex. Neuroscience 129:179–185. 10.1016/j.neuroscience.2004.08.005 15489040

[B25] Isaacson J, Solis J, Nicoll R (1993) Local and diffuse synaptic actions of GABA in the hippocampus. Neuron 10:165–175. 10.1016/0896-6273(93)90308-E 7679913

[B26] Ivanina T, Blumenstein Y, Shistik E, Barzilai R, Dascal N (2000) Modulation of L-type Ca2+ channels by G*βγ* and calmodulin via interactions with N and C termini of *α*1C. J Biol Chem 275:39846–39854. 10.1074/jbc.M005881200 10995757

[B27] Jedrzejewska-Szmek J, Luczak V, Abel T, Blackwell KT (2017) *β*-Adrenergic signaling broadly contributes to LTP induction. PLoS Comput Biol 13:e1005657. 10.1371/journal.pcbi.1005657 28742159 PMC5546712

[B28] Johnson KA, Lovinger DM (2016) Presynaptic g protein-coupled receptors: gatekeepers of addiction? Front Cell Neurosci 10:264. 10.3389/fncel.2016.00264 27891077 PMC5104741

[B29] Kaczmarek LK, Zhang Y (2017) Kv3 channels: enablers of rapid firing, neurotransmitter release, and neuronal endurance. Physiol Rev 97:1431–1468. 10.1152/physrev.00002.2017 28904001 PMC6151494

[B30] Kohl MM, Paulsen O (2010) The roles of GABAB receptors in cortical network activity. Adv Pharmacol 58:205–229. 10.1016/S1054-3589(10)58009-8 20655484

[B31] Kupferschmidt DA, Lovinger DM (2015) Inhibition of presynaptic calcium transients in cortical inputs to the dorsolateral striatum by metabotropic GABAB and mGlu2/3 receptors. J Physiol 593:2295–2310. 10.1113/JP270045 25781000 PMC4457193

[B32] Lehmann A, et al. (2009) (R)-(3-amino-2-fluoropropyl) phosphinic acid (AZD3355), a novel GABAB receptor agonist, inhibits transient lower esophageal sphincter relaxation through a peripheral mode of action. J Pharmacol Exp Therap 331:504–512. 10.1124/jpet.109.153593 19648470

[B33] Li L, Zhou J, Sun H, Liu J, Wang H, Liu X, Wang C (2020) A computational model to investigate GABA-activated astrocyte modulation of neuronal excitation. Comput Math Methods Med 2020:8750167. 10.1155/2020/8750167 33014120 PMC7512075

[B34] Lorenzon NM, Foehring RC (1995) Characterization of pharmacologically identified voltage-gated calcium channel currents in acutely isolated rat neocortical neurons. I. Adult neurons. J Neurophysiol 73:1430–1442. 10.1152/jn.1995.73.4.1430 7643158

[B35] Lubitz T, Hahn J, Bergmann FT, Noor E, Klipp E, Liebermeister W (2016) SBtab: a flexible table format for data exchange in systems biology. Bioinformatics 32:2559–2561. 10.1093/bioinformatics/btw179 27153616 PMC4978929

[B36] Mäki-Marttunen T, Halnes G, Devor A, Metzner C, Dale AM, Andreassen OA, Einevoll GT (2018) A stepwise neuron model fitting procedure designed for recordings with high spatial resolution: application to layer 5 pyramidal cells. J Neurosci Methods 273:264–283. 10.1016/j.jneumeth.2017.10.007 PMC5705475 28993204

[B37] Mäki-Marttunen T, Iannella N, Edwards AG, Einevoll GT, Blackwell KT (2020) A unified computational model for cortical post-synaptic plasticity. Elife 9:e55714. 10.7554/eLife.55714 32729828 PMC7426095

[B38] Mäki-Marttunen T, Mäki-Marttunen V (2022) Excitatory and inhibitory effects of HCN channel modulation on excitability of layer V pyramidal cells. PLoS Comput Biol 18:e1010506. 10.1371/journal.pcbi.1010506 36099307 PMC9506642

[B39] Manz KM, Baxley AG, Zurawski Z, Hamm HE, Grueter BA (2019) Heterosynaptic GABAB receptor function within feedforward microcircuits gates glutamatergic transmission in the nucleus accumbens core. J Neurosci 39:9277–9293. 10.1523/JNEUROSCI.1395-19.2019 31578230 PMC6867813

[B40] McDougal RA, Hines ML, Lytton WW (2013) Reaction-diffusion in the neuron simulator. Front Neuroinform 7:28. 10.3389/fninf.2013.00028 24298253 PMC3828620

[B41] Migliore M, Cook E, Jaffe D, Turner D, Johnston D (1995) Computer simulations of morphologically reconstructed ca3 hippocampal neurons. J Neurophysiol 73:1157–1168. 10.1152/jn.1995.73.3.1157 7608762

[B42] Oláh S, Komlosi G, Szabadics J, Varga C, Toth E, Barzo P, Tamas G (2007) Output of neurogliaform cells to various neuron types in the human and rat cerebral cortex. Front Neural Circ 1:84. 10.3389/neuro.04.004.2007 PMC2526278 18946546

[B43] Oláh S, Füle M, Komlósi G, Varga C, Báldi R, Barzó P, Tamás G (2009) Regulation of cortical microcircuits by unitary GABA-mediated volume transmission. Nature 461:1278–1281. 10.1038/nature08503 19865171 PMC2771344

[B44] Olpe HR, Steinmann MW, Greiner K, Pozza MF (1994) Contribution of presynaptic GABA-B receptors to paired-pulse depression of GABA responses in the hippocampus. Naunyn-Schmiedeberg’s Arch Pharmacol 349:473–477. 10.1007/BF00169135 8065460

[B45] Ostrovskaya O, Xie K, Masuho I, Fajardo-Serrano A, Lujan R, Wickman K, Martemyanov KA (2014) RGS7/G*β*5/R7BP complex regulates synaptic plasticity and memory by modulating hippocampal GABABR-GIRK signaling. Elife 3:e02053. 10.7554/eLife.02053 24755289 PMC3988575

[B46] Palmer LM, Schulz JM, Murphy SC, Ledergerber D, Murayama M, Larkum ME (2012) The cellular basis of GABAB-mediated interhemispheric inhibition. Science 335:989–993. 10.1126/science.1217276 22363012

[B47] Park A, Hoffman K, Keller A (2014) Roles of GABAA and GABAB receptors in regulating thalamic activity by the zona incerta: a computational study. J Neurophysiol 112:2580–2596. 10.1152/jn.00282.2014 25143541 PMC4233275

[B48] Pérez-Garci E, Gassmann M, Bettler B, Larkum ME (2006) The GABAB1b isoform mediates long-lasting inhibition of dendritic Ca2+ spikes in layer 5 somatosensory pyramidal neurons. Neuron 50:603–616. 10.1016/j.neuron.2006.04.019 16701210

[B49] Pérez-Garci E, Larkum ME, Nevian T (2013) Inhibition of dendritic Ca2+ spikes by GABAB receptors in cortical pyramidal neurons is mediated by a direct Gi/o-*βγ*-subunit interaction with Cav1 channels. J Physiol 591:1599–1612. 10.1113/jphysiol.2012.245464 23184512 PMC3624841

[B50] Price CJ, Cauli B, Kovacs ER, Kulik A, Lambolez B, Shigemoto R, Capogna M (2005) Neurogliaform neurons form a novel inhibitory network in the hippocampal ca1 area. J Neurosci 25:6775–6786. 10.1523/JNEUROSCI.1135-05.2005 16033887 PMC6725364

[B51] Santos JP, Pajo K, Trpevski D, Stepaniuk A, Eriksson O, Nair AG, Keller D, Hellgren Kotaleski J, Kramer A (2022) A modular workflow for model building, analysis, and parameter estimation in systems biology and neuroscience. Neuroinformatics 20:241–259. 10.1007/s12021-021-09546-3 34709562 PMC9537196

[B52] Scanziani M, Salin PA, Vogt KE, Malenka RC, Nicoll RA (1997) Use-dependent increases in glutamate concentration activate presynaptic metabotropic glutamate receptors. Nature 385:630–634. 10.1038/385630a0 9024660

[B53] Scanziani M (2000) GABA spillover activates postsynaptic GABAB receptors to control rhythmic hippocampal activity. Neuron 25:673–681. 10.1016/S0896-6273(00)81069-7 10774734

[B54] Schulz JM, Kay JW, Bischofberger J, Larkum ME (2021) GABA B receptor-mediated regulation of dendro-somatic synergy in layer 5 pyramidal neurons. Front Cell Neurosci 15:718413. 10.3389/fncel.2021.718413 34512268 PMC8425515

[B55] Stegen M, Kirchheim F, Hanuschkin A, Staszewski O, Veh RW, Wolfart J (2012) Adaptive intrinsic plasticity in human dentate gyrus granule cells during temporal lobe epilepsy. Cereb Cortex 22:2087–2101. 10.1093/cercor/bhr294 22038909

[B56] Traub RD, Jefferys J, Miles R, Whittington MA, Tóth K (1994) A branching dendritic model of a rodent CA3 pyramidal neurone. J Physiol 481:79–95. 10.1113/jphysiol.1994.sp020420 7853251 PMC1155867

[B57] Trubetskoy V, et al. (2022) Mapping genomic loci implicates genes and synaptic biology in schizophrenia. Nature 604:502–508. 10.1038/s41586-022-04434-5 35396580 PMC9392466

[B58] Tureček R, Melichar A, Králíková M, Hrušková B (2023) The role of GABAB receptors in the subcortical pathways of the mammalian auditory system. Front Endocrinol 14:1195038. 10.3389/fendo.2023.1195038 PMC10456889 37635966

[B59] Turi GF, et al. (2019) Vasoactive intestinal polypeptide-expressing interneurons in the hippocampus support goal-oriented spatial learning. Neuron 101:1150–1165. 10.1016/j.neuron.2019.01.009 30713030 PMC6428605

[B60] Wang XJ (1999) Synaptic basis of cortical persistent activity: the importance of NMDA receptors to working memory. J Neurosci 19:9587–9603. 10.1523/JNEUROSCI.19-21-09587.1999 10531461 PMC6782911

[B61] Wilkinson MD, et al. (2016) The fair guiding principles for scientific data management and stewardship. Sci Data 3:1–9. 10.1038/sdata.2016.18 PMC4792175 26978244

[B62] Yim MY, Hanuschkin A, Wolfart J (2015) Intrinsic rescaling of granule cells restores pattern separation ability of a dentate gyrus network model during epileptic hyperexcitability. Hippocampus 25:297–308. 10.1002/hipo.22373 25269417

[B63] Zhong H, Wade SM, Woolf PJ, Linderman JJ, Traynor JR, Neubig RR (2003) A spatial focusing model for G protein signals: regulator of G protein signaling (RGS) protein-mediated kinetic scaffolding. J Biol Chem 278:7278–7284. 10.1074/jbc.M208819200 12446706

